# An Ultra-Lightweight Fish Detection Model for Real-Time Aquatic Animal Monitoring on Embedded Platforms

**DOI:** 10.3390/ani16172640

**Published:** 2026-08-23

**Authors:** Hanyu Zhang, Zhongde Zhang, Weiping Liu

**Affiliations:** 1 School of Technology, Beijing Forestry University, Beijing 100083, China; zhanyyu@bjfu.edu.cn (H.Z.); zhongde@bjfu.edu.cn (Z.Z.); 2 Key Lab of State Forestry Administration for Forestry Equipment and Automation, Beijing 100083, China; 3 Beijing Laboratory of Urban and Rural Ecological Environment, Beijing Municipal Education Commission, Beijing 100083, China; 4 Research Center for Intelligent Forestry, Beijing 100083, China

**Keywords:** fish monitoring, underwater object detection, aquaculture, edge computing, lightweight deep learning, animal welfare, biodiversity assessment

## Abstract

Continuous, non-invasive fish monitoring supports aquatic animal management, biodiversity assessment, and sustainable aquaculture, but embedded deployment requires a careful balance among accuracy, speed, memory, and computation under visually degraded underwater conditions. We developed ULFD-YOLO, an ultra-lightweight detector derived from YOLOv11n through coordinated redesign of the backbone, neck, and detection head. The model combines a custom convolutional MobileNetV4-tiny backbone, a hypergraph-based multi-scale fusion neck, and a lightweight MBConv head with channel attention. Experiments were conducted on Fish-BJ, an in-house dataset of 3402 images covering 21 species-informed aquarium-fish detection categories, and on a deliberately difficult 1180-image WildFish subset after dataset-specific training. On Fish-BJ, ULFD-YOLO achieved 0.960 mAP@0.5 and 0.732 mAP@0.5:0.95 with 1.3 M parameters, 2.6 GFLOPs, and a 3.0 MB model file, reducing parameters and computation by 50.0% and 58.7% relative to YOLOv11n. Bootstrap resampling yielded 95% confidence intervals of 0.946–0.973 and 0.638–0.821 for the two metrics, respectively. The model achieved 0.803 mAP@0.5 on WildFish and 19–24 FPS at 448 × 640 on a Jetson Orin Nano under its 15 W nvpmodel power mode. These results establish a practical accuracy–efficiency trade-off for embedded fish monitoring rather than peak localization accuracy.

## Simple Summary

Monitoring fish without handling them can support biodiversity conservation, animal welfare, ecosystem assessment, and sustainable aquaculture. Camera-based monitoring reduces disturbance, but many detection models require computers that are too large or power-hungry for widespread use beside ponds, tanks, cages, or other aquatic observation sites. We developed an ultra-lightweight fish-detection model designed for small, low-power embedded devices. The model occupies only 3.0 megabytes and processes 19–24 frames per second on an embedded platform while retaining useful detection accuracy. It was evaluated using an in-house aquarium dataset containing challenging conditions such as variable illumination, turbidity, occlusion, and small or distant fish, and was also trained and evaluated on a difficult subset of a public fish-image dataset. The results show that real-time fish detection can be achieved with substantially lower computational demand. This approach may provide a practical visual-monitoring foundation for future applications in fish-population assessment, feeding management, animal-welfare monitoring, and aquatic conservation.

## 1. Introduction

Accurate and efficient underwater fish detection is important for ocean engineering, aquatic animal monitoring, fishery resource assessment, ecological inspection, and intelligent aquaculture management [1]. In intensive and semi-intensive farming systems, real-time information on fish abundance, spatial distribution, feeding behavior, and health status is essential for management decisions and sustainable production [2,3]. However, traditional approaches, including manual visual inspection and periodic physical sampling, are labor-intensive, time-consuming, and potentially disruptive to fish and their rearing environment [4,5]. The growing scale of modern aquaculture has therefore increased the demand for automated, non-invasive, and continuous visual monitoring systems that can operate with limited human intervention [6].

It should be emphasized that continuous and non-invasive observation is a general property of camera-based monitoring rather than a unique benefit of lightweight models: a camera connected to a shore-based server running a large detector would also provide continuous, non-invasive imaging. The distinctive contribution of an ultra-lightweight detector is therefore not non-invasiveness itself, but the ability to lower the cost and complexity of on-site deployment. In practice, camera-based monitoring involves a concrete engineering trade-off between two deployment strategies. Wired camera nodes can stream video to a central server for inference, but they require cabling, waterproof connectors, and periodic lens cleaning and maintenance. Battery- or solar-powered wireless nodes avoid cabling but incur recurring energy and maintenance costs, which grow rapidly as the number of nodes increases. On-device inference with an ultra-lightweight detector reduces the volume of data that must be transmitted, lowers the compute and power required at each node, and enables local decision-making, thereby reducing both bandwidth demand and the frequency of physical maintenance. These operational costs are often underestimated in machine-learning studies, yet they dominate the total cost of ownership in real aquaculture deployments. The present work is motivated primarily by this deployment-cost consideration rather than by non-invasiveness alone.

Deep learning-based computer vision has become a promising tool for automated fish detection in aquaculture facilities [7,8]. Among current detection frameworks, single-stage detectors from the YOLO family are widely used in aquaculture-related tasks because they provide a favorable balance between inference speed and detection accuracy [9,10,11]. Earlier studies showed that YOLOv3 and YOLOv4 can be adapted for fish detection and counting under farming conditions [12,13]. More recent models have incorporated attention mechanisms, multi-scale feature enhancement, and hybrid CNN-Transformer designs to address low contrast, uneven illumination, turbidity, and color distortion in underwater imagery [14,15]. Although these improvements enhance detection robustness, they often increase model complexity and computational cost, limiting deployment on the embedded devices commonly used in aquaculture facilities [16,17].

This deployment constraint is particularly important in aquaculture engineering. Underwater camera nodes, remotely operated vehicles, and edge computing units used in fish farms usually provide much less processing power, memory, and energy than laboratory-grade GPU servers [18]. Consequently, practical monitoring systems require lightweight detectors that can perform real-time inference on embedded platforms while maintaining the accuracy needed for reliable stock assessment, behavior monitoring, and animal welfare evaluation [17].

Existing lightweight detection strategies can be grouped into compact architecture design and post-training model compression. Compact networks, including MobileNet, ShuffleNet [19,20], and GhostNet [21,22], reduce parameters and computation through depthwise separable convolutions, channel shuffling, or feature reuse. Compression techniques such as pruning, quantization, and knowledge distillation can further reduce model size and latency [23,24]. However, these generic strategies do not fully account for the visual degradation typical of underwater aquaculture imagery. Fish are often small, partially occluded by structures or neighboring individuals, and embedded in cluttered backgrounds containing aquatic vegetation, suspended particles, biofilm, and variable lighting [25,26]. Under aggressive compression, performance losses are often concentrated in precisely these difficult cases, which are critical for accurate population assessment and behavioral monitoring [27,28].

This limitation defines the central problem addressed in this study. For aquaculture monitoring, reducing parameters or floating point operations (FLOPs) alone is not sufficient. Lightweight design must also preserve multi-scale feature representation and discriminative capacity under adverse underwater imaging conditions [29]. The key challenge is therefore to obtain an extremely compact detector while retaining robust performance for small, visually ambiguous, and partially occluded fish targets in practical aquaculture scenarios [27].

To address this challenge, we propose ULFD-YOLO, an ultra-lightweight fish detection model redesigned from YOLOv11n [30] for embedded aquaculture monitoring platforms. Rather than applying isolated compression operations, ULFD-YOLO jointly redesigns the backbone, neck, and detection head to optimize both computational efficiency and detection capability. A custom MobileNetV4-tiny backbone derived from the convolutional MobileNetV4 design [31] provides an efficient feature-extraction foundation for resource-constrained hardware. A hypergraph-based feature-fusion neck (Hyper-YOLO) [32] enhances higher-order multi-scale semantic interactions, improving the representation of small fish targets whose fine visual cues may be lost during compression. In addition, a lightweight Detect_MBConv head, built on the MBConv principle [33] and equipped with channel attention, reduces prediction-stage cost while preserving discrimination against cluttered backgrounds. Through this coordinated design, ULFD-YOLO aims to provide a deployable balance between accuracy and efficiency for real-time fish monitoring.

We note that MobileNetV4, hypergraph-based feature fusion, and the MBConv/SE principle are individually established techniques. The contribution of this study is therefore not the invention of a new atomic module, but a problem-driven co-design in which the three stages are matched to the specific failure modes of ultra-lightweight underwater fish detection. Concretely, aggressive backbone compression can erode small-target and occluded-object representation; we pair a custom convolutional MobileNetV4-tiny backbone with a hypergraph neck intended to recover higher-order cross-scale information and with an SE-equipped MBConv head intended to preserve channel discrimination while remaining lightweight. The full factorial ablation shows that the complete configuration provides the lowest-FLOP result among the tested variants while retaining 0.960 mAP@0.5. Compared with generic lightweight YOLO variants that compress uniformly, ULFD-YOLO redistributes cost toward the stages that most affect underwater fish detection, and the resulting accuracy–computation trade-off is quantified rather than assumed.

The main contributions of this study are as follows:

- First, we propose ULFD-YOLO, an ultra-lightweight fish detector for resource-constrained aquaculture monitoring platforms. By jointly redesigning the backbone, neck, and detection head, the model reduces parameter count by 50.0% and computational cost by 58.7% relative to YOLOv11n while maintaining competitive detection accuracy.
- Second, we develop a task-oriented lightweight architecture for underwater fish imagery. The MobileNetV4-tiny backbone provides efficient feature extraction, the hypergraph-based neck strengthens multi-scale representation of small and partially occluded fish, and the MBConv-based detection head with channel attention supports efficient prediction and background suppression.
- Third, extensive experiments on a private 21-category aquarium fish dataset (Fish-BJ) and on a public second dataset (WildFish) after dataset-specific training support the proposed design. Deployment on an NVIDIA Jetson Orin Nano further demonstrates real-time model-inference feasibility, reaching 19–24 FPS under the Jetson Orin Nano’s 15 W nvpmodel power mode.

## 2. Materials and Methods

### 2.1. Dataset Description

#### 2.1.1. Fish-BJ Dataset

The primary experiments were conducted on Fish-BJ, an in-house underwater fish image dataset independently constructed and annotated by our research team. All images were captured by our team at Beijing Aquarium, China. The dataset was built entirely in-house and was not obtained, derived, or adapted from a third-party dataset. It was created to evaluate detection models under visually challenging monitoring conditions. Although the dataset was not collected from commercial freshwater aquaculture facilities, the imaging conditions reflect several challenges encountered in practical underwater fish monitoring and aquaculture-like environments, including variable illumination, water turbidity, complex backgrounds, fish posture variation, scale changes, dense fish distributions, and partial occlusion. The 21 labels are species-informed detection categories represented by widely used common names. Ground-truth bounding boxes were manually annotated using LabelImg and subsequently checked by multiple annotators to improve annotation consistency.

The acquisition setup was as follows. Images were captured with a Canon EOS R6 Mark II camera (Canon Inc., Tokyo, Japan) operated in air, positioned outside the exhibit tanks and imaging the fish through the viewing panel; the camera was not submerged at any point. The distance from the camera to the imaged fish ranged from approximately 1 to 5 m, and illumination was provided solely by the facility’s standard artificial exhibit lighting, without flash or supplementary illumination. Images were captured at the camera’s native resolution of 6000 × 4000 pixels and were subsequently resized to 640 × 640 pixels for training and evaluation. Because no camera was placed in the water, quantities that are defined only for a submerged camera, such as camera submersion depth, water depth at the camera, and underwater-housing or port characteristics, are not applicable to this dataset and were not recorded. The exhibit water is professionally filtered and maintained by the facility; quantitative turbidity and water-quality measurements were not performed, and the water condition is therefore described qualitatively rather than in quantitative terms.

Although the camera was not submerged, the optical path from each fish to the lens traverses a water column whose length varies with the position of the animal within the tank, and the resulting images exhibit degradations that affect detector performance in underwater scenes: wavelength-dependent attenuation and blue-green color cast, scattering by suspended particles, variable turbidity, non-uniform artificial illumination and panel glare, background clutter from tank structures and aquatic vegetation, and dense multi-individual occlusion. Through-panel imaging does not, however, reproduce degradations specific to a submerged camera, including port refraction, lens biofouling, backscatter from an on-board light source, and low-light conditions at depth. This limitation is stated in Section 4.4. The architecture was also separately trained and evaluated on the WildFish benchmark, which contains genuine in-water imagery, to assess whether its accuracy–efficiency behavior can be reproduced under a different imaging regime.

Images were acquired under routine aquarium display conditions rather than in a controlled laboratory imaging setup. Therefore, the viewing angle, shooting distance, illumination intensity, background structure, and water clarity varied among tanks and acquisition sessions. These variations were retained intentionally because they help approximate the visual complexity of practical underwater monitoring scenarios. All images used for model training and evaluation were resized to 640 × 640 pixels to ensure consistency across detectors. The retained scenes include clear and moderately turbid water conditions, heterogeneous illumination, fish at different distances from the camera, and backgrounds containing tank structures, aquatic vegetation, suspended particles, and other visual interference.

Annotation followed a fixed protocol. All visible fish instances were annotated whenever their category identity and approximate spatial extent could be determined with reasonable confidence. Difficult instances—including motion-blurred, partially occluded, truncated, distant, low-contrast, and incompletely visible fish—were deliberately retained and annotated to the greatest extent possible because Fish-BJ was designed to preserve the visual complexity of real underwater monitoring environments. Tight axis-aligned bounding boxes were assigned on the basis of the discernible target extent. Instances that remained too ambiguous for reliable category assignment or localization after consensus review were excluded from the valid annotation target set. All annotations were reviewed by a second annotator, and disagreements were resolved through discussion until consensus was reached. This multi-step checking procedure was used to improve the reliability and consistency of the bounding-box annotations.

The original Fish-BJ image pool contained 10,581 candidate images covering 21 species-level fish categories, with per-category image counts ranging from approximately 360 to 600. The reduction to 3402 retained images did not result from removing all visually difficult samples. Images containing blur, turbidity, partial occlusion, truncation, distant targets, or incompletely visible fish were retained whenever at least one fish target could be annotated with reasonable confidence. Images were removed primarily when they contained no discernible fish target, when the available visual information was insufficient to assign any reliable category and bounding box after consensus review, when annotation records were invalid and could not be corrected, or when frames were near-duplicates generated during continuous acquisition. This procedure reduced non-informative and redundant material while preserving difficult but realistic monitoring scenes.

After this quality-control and annotation-filtering process, 3402 images with valid bounding-box annotations were retained for model development. Following a commonly used hold-out evaluation protocol in deep learning-based object detection studies, these images were divided into 2721 training images and 681 held-out evaluation images using an 8:2 split with a fixed random seed (seed = 42). The training subset was used for parameter updating. The held-out subset was used by the Ultralytics framework for its default validation-based best-checkpoint selection and was also used for reporting the final performance (see Section 2.3, where the consequences of this dual role are stated). The same fixed split was applied to all baseline detectors, ablation variants, and the proposed ULFD-YOLO model to ensure a controlled comparison.

The retained dataset preserves diverse fish poses, including frontal, lateral, and ventral views, as well as a wide range of target scales from close-range individuals to distant small fish. It also includes complex underwater scenes with multiple targets, dense fish distributions, mutual occlusion, background interference, suspended particles, and heterogeneous illumination. These characteristics make Fish-BJ suitable for evaluating lightweight fish detectors under realistic underwater monitoring conditions and for approximating visual challenges that may occur in aquaculture-oriented deployment scenarios.

To ensure that the split was principled rather than arbitrary, the dataset partition was constructed in a category-stratified and near-duplicate-aware manner. Category stratification preserved the per-category image distribution in the training and held-out evaluation subsets, so that the evaluation subset remained representative of all 21 detection categories rather than being skewed toward a subset of more frequent categories. In addition, near-duplicate frames originating from continuous image acquisition were identified during screening and removed or retained within the same subset when identifiable, thereby reducing the risk that visually near-identical frames would appear in both the training and evaluation subsets. The same fixed split was applied to all detectors, ablation variants, and the proposed model. The post-filtering image counts and bounding-box counts are reported in Table 1.

#### 2.1.2. WildFish Dataset

To evaluate whether the accuracy–efficiency behavior of ULFD-YOLO can be reproduced after dataset-specific training on a second dataset with different category composition and imaging conditions, additional experiments were conducted on the public WildFish dataset [34]. WildFish contains fish images from diverse natural aquatic environments and exhibits substantial variation in appearance, water quality, illumination, and background structure. Ten fish categories were purposively selected to construct an exactly 1180-image difficult subset, comprising 944 training images and 236 held-out evaluation images. The selected images generally contain a single focal fish category, and no image contains more than one of the ten selected categories. The selection was purposive rather than random: the chosen categories and scenes include low contrast, cryptic or disruptive coloration, small apparent size, frequent occlusion, and cluttered natural backgrounds. Accordingly, the absolute results should be interpreted as performance on a difficult WildFish subset rather than as an estimate of average performance on the full benchmark. All compared detectors were trained on the same 944-image WildFish training split and evaluated on the same 236-image held-out split.

Because WildFish was originally released as a fine-grained recognition benchmark rather than a detection benchmark, the selected images did not contain bounding-box annotations. For the detection experiments in this study, all discernible fish instances belonging to the focal image category were manually annotated with axis-aligned bounding boxes using LabelImg. When multiple individuals of the same category were visible, each discernible individual was annotated. Because no selected image contained more than one of the ten target categories, no multi-category label ambiguity occurred within an image. The same tight-box and second-annotator review protocol used for Fish-BJ was applied. The selected subset contains 1269 annotated fish instances across the ten categories. The WildFish experiment is therefore an evaluation on a second dataset after dataset-specific training, not a zero-shot transfer test from Fish-BJ.

Ethics statement: data collection for Fish-BJ involved only passive imaging of fish in their existing housing at Beijing Aquarium, carried out from outside the exhibit tanks through the viewing panels; no camera or other equipment was placed in the water. No handling, transport, confinement, tagging, or other invasive procedures were performed on the animals for the purpose of this study, and the imaging did not alter the normal husbandry of the facility. The WildFish images were obtained from the publicly released dataset.

### 2.2. ULFD-YOLO Model Architecture

#### 2.2.1. Overview

**Figure 1:**
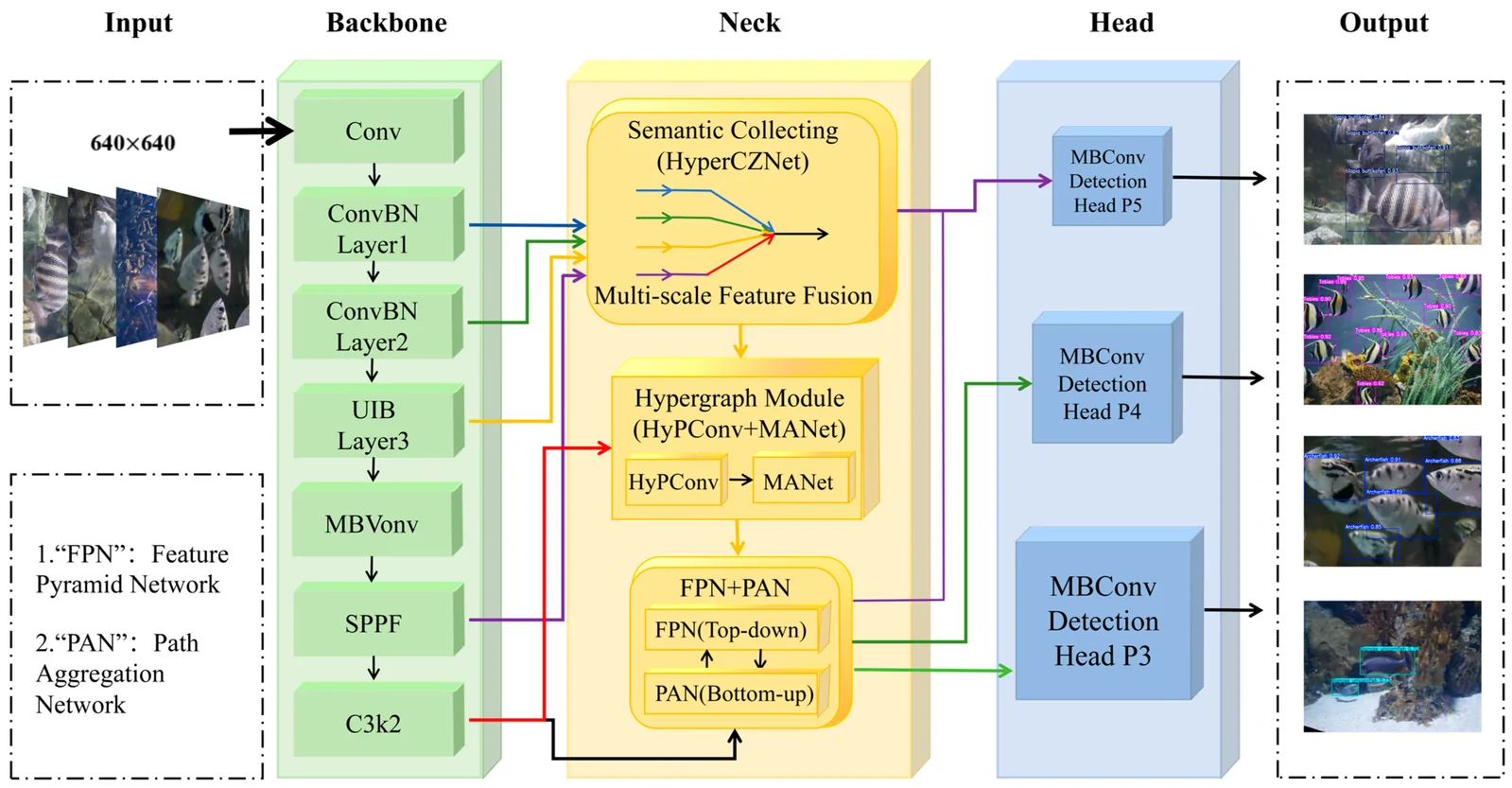
Overall architecture of the proposed ULFD-YOLO network. The model contains three redesigned components: a custom convolutional MobileNetV4-tiny backbone for efficient feature extraction, a Hyper-YOLO neck for higher-order multi-scale feature fusion, and a Detect_MBConv head for lightweight and discriminative prediction. Colored blocks indicate the key modified modules relative to the YOLOv11n baseline.(Ar row colors distinguish feature streams originating from different backbone stages, whereas black arrows indicate sequential processing and output connections.)

The overall architecture of ULFD-YOLO is shown in Figure 1. Starting from the YOLOv11n baseline [30], the proposed detector is redesigned through coordinated modifications of the backbone, neck, and detection head to improve deployment efficiency on embedded hardware while maintaining reliable underwater fish representation.

The original backbone is replaced by a custom MobileNetV4-tiny configuration derived from the convolutional MobileNetV4 design (Section 2.2.3) to reduce feature-extraction cost and parameter redundancy under the computational constraints of embedded aquaculture devices. The feature-fusion pathway is reconstructed using a hypergraph-based neck (Section 2.2.4), which strengthens higher-order multi-scale semantic interactions and improves the representation of fish targets with variable size and occlusion. Finally, a lightweight Detect_MBConv head (Section 2.2.5) is introduced to reduce prediction-stage cost while preserving channel discrimination.

These three modifications form a coordinated lightweight redesign pipeline. The backbone establishes an efficiency-oriented foundation, the neck compensates for representational loss caused by aggressive compression, and the detection head enables efficient final prediction under strict resource constraints. The resulting design achieves a practical balance between detection accuracy and deployment efficiency for real-time monitoring on embedded aquaculture platforms.

#### 2.2.2. YOLOv11 Baseline Architecture

YOLOv11n was selected as the baseline detector because it offers a competitive balance between accuracy and inference speed among current single-stage frameworks [30]. Its architecture follows the standard backbone–neck–head pipeline: the backbone extracts hierarchical visual features, the neck aggregates multi-scale semantic information, and the detection head generates final localization and classification predictions.

Although YOLOv11n is relatively compact (2.6 M parameters and 6.3 GFLOPs), three structural characteristics limit direct deployment on aquaculture monitoring hardware. First, the backbone retains computational redundancy under the strict power and memory budgets of underwater camera nodes and portable monitoring units. Second, the PANet-based neck mainly relies on adjacent-layer feature propagation, which is insufficient for modeling the higher-order cross-scale dependencies needed to detect small or partially occluded fish in cluttered farming environments. Third, the detection head accounts for a non-negligible share of total computation, and its discriminative capacity under degraded underwater imaging conditions leaves room for improvement. These observations motivated the coordinated redesign described in Section 2.2.3, Section 2.2.4 and Section 2.2.5.

#### 2.2.3. MobileNetV4-Tiny Lightweight Backbone

MobileNetV4 is a hardware-aware lightweight architecture designed to balance representational capacity and inference latency across heterogeneous edge devices [31]. The following characteristics of the broader MobileNetV4 family motivate the convolutional configuration adapted in this study.

First, MobileNetV4 introduces the Universal Inverted Bottleneck (UIB), a flexible building block based on depthwise separable convolution (DSC). Unlike standard convolution, which couples spatial filtering and channel mixing into a single operation, its computational cost can be written as:

(1) $$\mathrm{Cos}t_{Standard}=H\times W\times K^{2}\times C_{in}\times C_{out}$$

DSC decomposes this operation into independent depthwise and pointwise stages:

(2) $$\mathrm{Cos}t_{DSC}=H\times W\times K^{2}\times C_{in}+H\times W\times C_{in}\times C_{out}$$

Compared with standard convolution, the relative computational cost of depthwise separable convolution is approximately *Cost_DSC_*/*Cost_Standard_* ≈ 1/*C_out_* + 1/*K*^2^. This means that depthwise separable convolution requires only a fraction of the computation used by standard convolution. Accordingly, the theoretical FLOP reduction ratio is 1 − (1/*C_out_* + 1/*K*^2^), and the corresponding speed-up factor can be approximated as [1/*C_out_* + 1/*K*^2^]^−1^. This decomposition substantially reduces computation, with the saving mainly determined by the kernel size K and the number of output channels *C_out_*.

Second, the broader MobileNetV4 family also includes Mobile Multi-Query Attention, which reduces memory traffic by sharing key and value projections across attention heads. This attention component is not used in the custom convolutional tiny configuration adopted in ULFD-YOLO; it is described here only to distinguish the selected configuration from the hybrid MobileNetV4 variants.

Third, MobileNetV4 supports NAS-driven model instantiation at multiple scales. In this study, MobileNetV4-tiny denotes a custom reduced convolutional configuration derived from the MobileNetV4 design; it is not one of the five officially named MobileNetV4 variants. The adopted configuration relies on pure-convolutional Universal Inverted Bottleneck modules and omits the heavier hybrid-attention components, thereby reducing parameters and computation while preserving high data reuse and low memory-access overhead.

Replacing the original YOLOv11n backbone with the custom MobileNetV4-tiny configuration is the first stage of the lightweight redesign. The backbone comparison results presented in Section 3.2.1 show that this modification reduces parameters from 2.6 M to 1.8 M (−30.8%), FLOPs from 6.3 G to 4.5 G (−28.6%), and model size from 5.5 MB to 3.9 MB, with a modest mAP@0.5 decrease from 0.98 to 0.95. This trade-off frees computational budget for the feature-fusion and detection-head components introduced in the subsequent stages.

**Figure 2:**
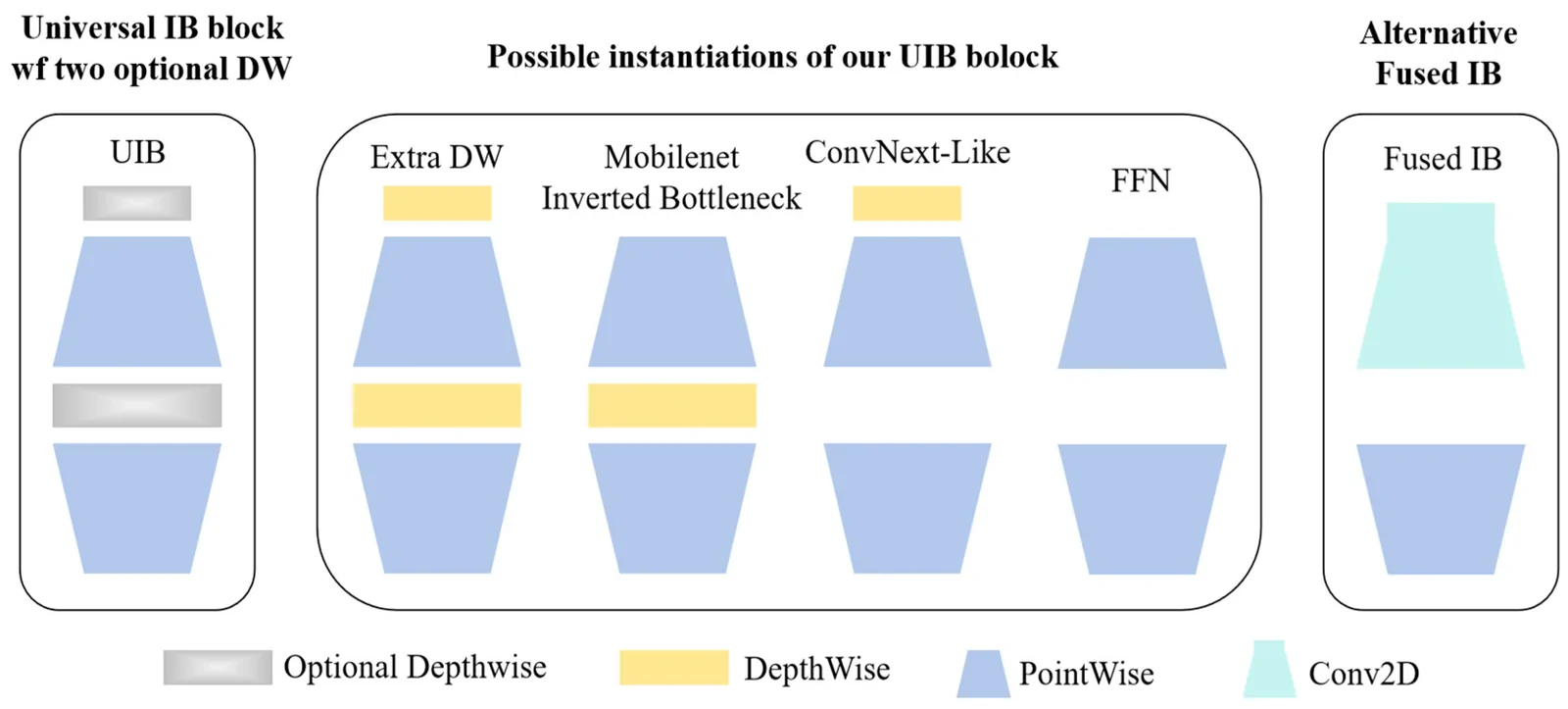
Detailed structure of the custom MobileNetV4-tiny backbone adopted in ULFD-YOLO. The backbone is derived from the convolutional MobileNetV4 design and is built from Universal Inverted Bottleneck modules with depthwise separable convolutions, enabling efficient hierarchical feature extraction with fewer parameters and lower computational cost than the original YOLOv11n backbone.

The detailed structure of the custom MobileNetV4-tiny backbone is shown in Figure 2.

#### 2.2.4. Hypergraph-Based Feature Fusion Neck

In underwater aquaculture environments, fish targets can vary greatly in scale, from small fry to full-grown individuals within the same field of view, and may be obscured by tank walls, net structures, neighboring fish, aquatic vegetation, and suspended particles. Conventional YOLO necks, such as PANet, mainly rely on adjacent-layer feature propagation and have limited ability to model higher-order cross-scale and cross-position semantic dependencies [35]. To address this limitation, ULFD-YOLO adopts the Hyper-YOLO neck, which incorporates hypergraph computation into multi-scale feature fusion [32]. Unlike ordinary graphs, in which each edge connects only two vertices, a hyperedge can connect multiple vertices simultaneously, allowing higher-order correlations among fish features distributed across spatial positions and semantic scales to be modeled.

**Figure 3:**
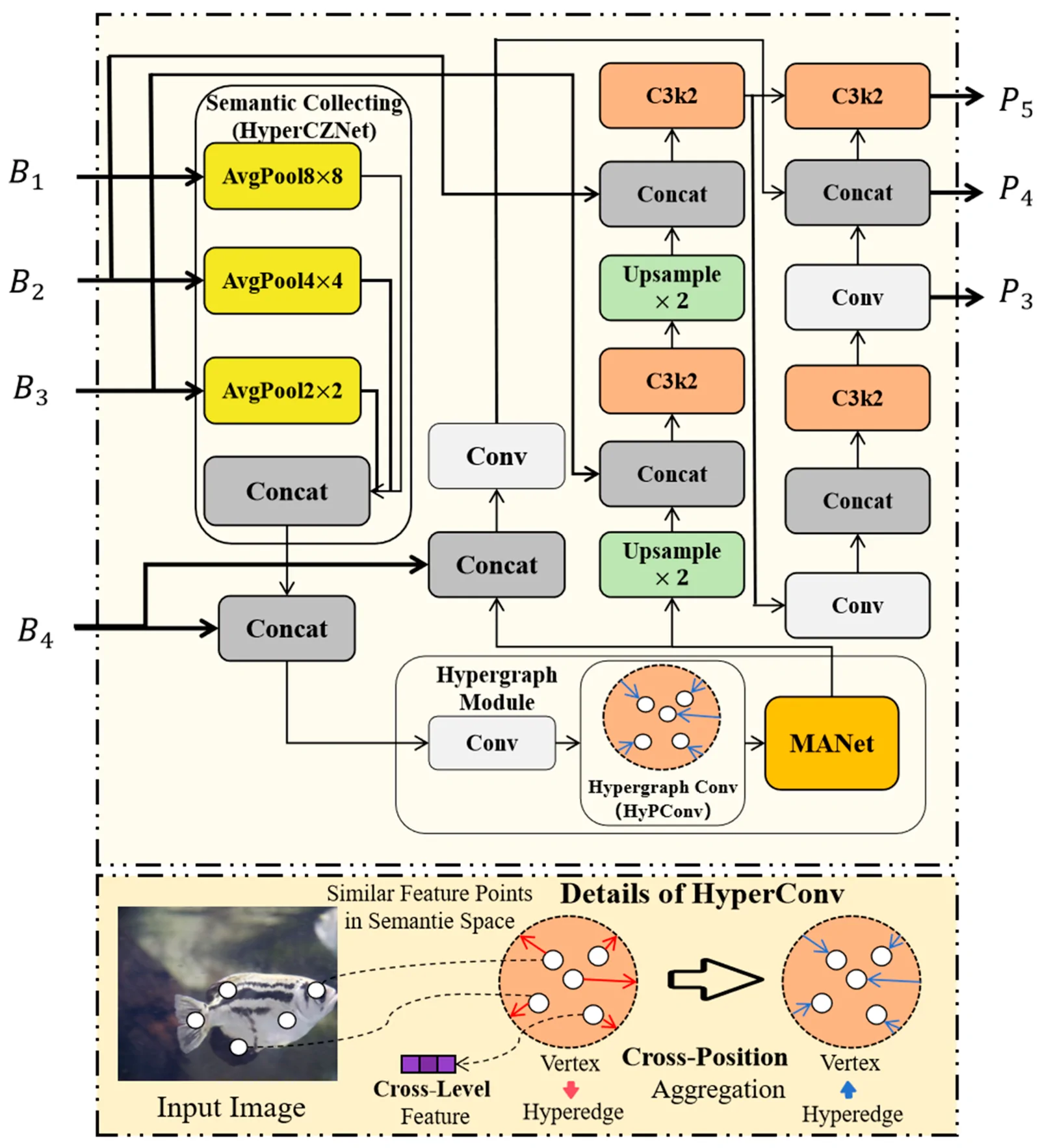
Architecture of the proposed Hyper-YOLO neck in ULFD-YOLO. Multi-scale features are aggregated through semantic collection, hypergraph computation, and semantic distribution, enhancing higher-order feature fusion for small and partially occluded fish in cluttered underwater scenes. (Black arrows indicate the main feature-flow paths, while the colored arrows in the HyperConv inset indicate hyperedge connections and aggregation directions, as defined in the inset legend.)

The architecture of the introduced Hyper-YOLO neck is shown in Figure 3.

The implementation uses HyperC2Net, which follows a three-stage Collect-Compute-Distribute pipeline. In the collection stage, feature maps from multiple backbone scales are aligned and aggregated into a unified semantic space. In the computation stage, a hypergraph is dynamically constructed by grouping semantically similar feature points, and hypergraph convolution performs cross-position aggregation:

(3) $$X^{\left(l+1\right)}=\sigma \left(D_{v}^{-\frac{1}{2}}HWD_{e}^{-1}H^{T}D_{v}^{-\frac{1}{2}}X^{\left(l\right)}\Theta^{\left(l\right)}\right)$$

where *X^(l)^* denotes the node features at layer l, *H* is the incidence matrix describing node-hyperedge membership, *W* is the diagonal hyperedge weight matrix, and *D_v_* and *D_e_* denote the node-degree and hyperedge-degree matrices used for symmetric normalization. Specifically, *D_v_* normalizes the contribution received by each node, whereas *D_e_* normalizes the aggregation over each hyperedge, preventing nodes or hyperedges with larger degrees from dominating the feature propagation. *Θ^(l)^* is the learnable transformation matrix. In the distribution stage, the enhanced representations are redistributed to their corresponding scales and refined through the Mixed Aggregation Network (MANet) and bottom-up C2f modules.

The hypergraph-based neck is intended to compensate for part of the representational loss introduced by the ultra-lightweight backbone. By modeling higher-order semantic interactions across positions and scales, it expands the effective perception range of the custom MobileNetV4-tiny backbone and can help recover subtle fish cues, especially those from small individuals weakened during compression. The component-level ablation results presented in Section 3.2.3 are consistent with this interpretation: adding Hyper-YOLO to the compressed backbone increases Recall from 0.900 to 0.920 and mAP@0.5:0.95 from 0.723 to 0.775, while the mechanism analysis in Section 4.2 provides a plausible explanation for the observed improvement.

It should be clarified that the Hyper-YOLO neck is a representation-enhancement component and is not itself a parameter-reduction mechanism. The component-level ablation results presented in Section 3.2.3 are consistent with this interpretation: when the neck is added on its own to the YOLOv11n baseline (the H-only configuration), the parameter count actually rises to 3.2 M. The overall reduction to 1.6 M parameters reported here is achieved because the neck is combined with the MobileNetV4-tiny backbone (the M+H configuration): the backbone is the source of the compression, whereas the neck restores the multi-scale representational capacity that the compressed backbone would otherwise lose. The wording in this section refers to the combined M+H model and should not be read as implying that the neck reduces parameters by itself.

#### 2.2.5. Detect_MBConv Lightweight Detection Head

To further improve inference efficiency on embedded aquaculture hardware, we introduce Detect_MBConv, a lightweight detection head based on the Mobile Inverted Bottleneck Convolution principle [33]. The module combines channel expansion, efficient spatial extraction, and adaptive channel attention in a compact prediction structure.

**Figure 4:**
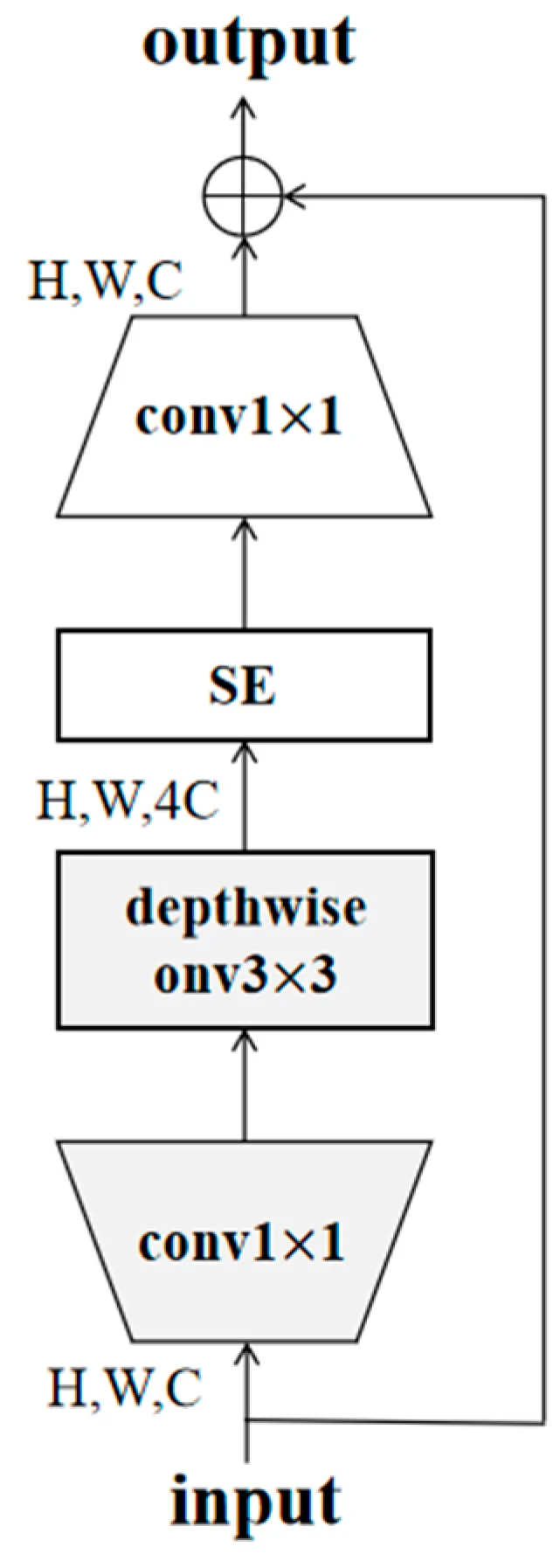
Architecture of the proposed Detect_MBConv detection head. The module uses a 1 × 1 pointwise convolution for channel expansion, a 3 × 3 depthwise convolution for spatial feature extraction with an embedded Squeeze-and-Excitation attention mechanism for adaptive channel reweighting, and a final 1 × 1 pointwise convolution for channel compression. Shortcut connections are incorporated to support feature reuse and stabilize gradient propagation.

The architecture of the introduced Detect_MBConv head is shown in Figure 4.

The detection head operates in two steps. First, a 1 × 1 pointwise convolution expands the channel dimension, followed by a 3 × 3 depthwise convolution for low-cost spatial feature extraction. A Squeeze-and-Excitation (SE) attention module is embedded at this stage to perform global average pooling and channel reweighting, thereby emphasizing fish-relevant channels while suppressing background interference from turbidity, suspended particles, and light scattering. The SE operation is expressed as:

(4) $$s=\sigma \left(W_{2}\delta \left(W_{1}z\right)\right)$$

where *z* is the channel descriptor obtained through global average pooling, *W*_1_ and *W*_2_ are the weights of two fully connected layers, *δ* is the ReLU activation function, and *σ* is the Sigmoid activation function. The output vector *s* represents the attention weight of each channel and is used to recalibrate the original feature maps.

This design offers three practical advantages for fish detection on resource-constrained aquaculture platforms. First, depthwise separable convolution substantially reduces parameters and computation relative to standard convolution. Second, the expand-then-compress MBConv structure preserves information flow while supporting efficient feature transformation. Third, the SE mechanism is intended to preserve channel discrimination while the detection head is compressed, helping limit the loss of fish-relevant information in cluttered underwater scenes.

The component-level ablation results presented in Section 3.2.3 are consistent with this interpretation: integrating Detect_MBConv yields the most computationally efficient complete configuration: 1.3 M parameters, 2.6 GFLOPs, and a 3.0 MB model size. These values correspond to reductions of 50.0% and 58.7% in parameters and FLOPs relative to YOLOv11n. The model retains 0.960 mAP@0.5, completing the lightweight redesign while preserving competitive detection performance for embedded aquaculture monitoring.

### 2.3. Training Configuration

All baseline and improved models were trained and evaluated under a controlled protocol. The hardware platform consisted of an AMD EPYC 9654 processor (Advanced Micro Devices, Inc., Santa Clara, CA, USA), with 30 CPU cores allocated to the experimental environment, 128.8 GB RAM, and an NVIDIA RTX PRO 6000 GPU (NVIDIA Corporation, Santa Clara, CA, USA) with 96.0 GB memory. The software environment used Linux, Python 3.9.24, PyTorch 2.8.0, and CUDA 13.0. On Fish-BJ, all models were trained only on the 2721-image training subset and evaluated on the same 681-image held-out subset. No held-out image was used for parameter updating. Because a large number of baseline detectors and ablation variants were compared, a single fixed hold-out protocol was adopted instead of k-fold cross-validation. The split was category-stratified, near-duplicate-aware, generated with a fixed dataset-partition seed of 42, and applied identically to all models. The same experimental design was applied to WildFish using its fixed 944/236 training/evaluation split.

For the YOLO-family models and all ablation variants, the input image resolution was fixed at 640 × 640 pixels, and training was conducted for 300 epochs with a batch size of 512. The stochastic gradient descent (SGD) optimizer was used with the framework-default learning-rate schedule and weight-decay settings. The Fish-BJ partition was generated once using dataset-partition seed 42 and remained unchanged throughout all experiments. The primary training run used model-training seed 0, and two additional runs used model-training seeds 1 and 42. Apart from the training seed, the data partition, initialization policy, hyperparameters, augmentation settings, and evaluation subset were held fixed across the three runs. Mosaic augmentation was enabled during early training and disabled during the final 10 epochs (close_mosaic = 10).

All YOLO-family detectors were initialized from publicly released pretrained weights associated with the corresponding implementation and were fully fine-tuned; no backbone was frozen. For Faster R-CNN, DETR, and EfficientDet, the architecture-specific default optimizer, learning-rate schedule, loss functions, assignment strategy, and post-processing of the respective reference implementation were retained. Their batch sizes were adjusted only to fit the available GPU memory, and the epoch budget was standardized to 300. No exhaustive architecture-specific hyperparameter search was performed; these high-complexity models are therefore retained as equal-budget reference points rather than optimized upper bounds. For NMS-based YOLO detectors, a confidence threshold of 0.001 was used as the low prediction-retention threshold during validation, and an NMS IoU threshold of 0.7 was applied. mAP was computed from the full precision–recall curve, whereas the reported Precision, Recall, and F1-score are the framework-reported summary operating-point values. Architecture-specific set-based or end-to-end NMS-free post-processing was retained for DETR and for YOLO variants whose reference implementation provides NMS-free inference, including YOLOv10 and YOLO26. The primary controlled comparison is restricted to nano-scale YOLO-family models trained within the common framework.

The held-out Fish-BJ subset was used both by the Ultralytics framework for default validation-based best-checkpoint selection and for reporting final performance. The framework-generated best.pt checkpoint was used for the primary results. The subset is therefore not a fully independent test set, and the absolute metrics may contain a small optimistic bias associated with checkpoint selection. Applying the same framework rule to every model reduces procedural differences, although model-specific sensitivity to validation-based selection cannot be excluded. As a sensitivity check, the final-epoch ULFD-YOLO checkpoint (last.pt) was also evaluated on the same subset. Image-level bootstrap confidence intervals, paired model comparison, and repeated-seed analysis are described in Section 2.4. To support transparency and reproducibility, a public research repository is available at https://github.com/999er/ULFD-YOLO (accessed on 22 June 2026). At the time of submission, the repository provides the exact Fish-BJ training/evaluation split list, per-category and per-split annotation statistics, the ULFD-YOLO model configuration and custom module implementation, training and validation scripts, prediction-export utilities, the stored held-out predictions, the outputs from the three training seeds, the statistical-analysis scripts, and the detection annotations created for the selected WildFish subset.

### 2.4. Evaluation Metrics

Two complementary groups of metrics were used to evaluate detection performance and deployment efficiency. Detection performance was assessed using Precision (P), Recall (R), F1-score, and mAP@0.5. The main metrics are defined as:

(5) $$Precision=\frac{TP}{TP+FP}$$

(6) $$Recall=\frac{TP}{TP+FN}$$

(7) $$mAP@0.5=\frac{1}{c}\sum_{k=1}^{c}AP_{k}@0.5$$

(8) $$F1-score=\frac{2}{\frac{1}{Precision}+\frac{1}{Reacll}}=\frac{2\times Precision\times Recall}{Precision+Recall}$$

*Precision* measures the proportion of correct positive predictions among all positive predictions. *Recall* represents the fraction of valid fish targets successfully detected, which is particularly relevant to downstream stock-assessment and population-monitoring applications. The *F1-score* provides a balanced assessment of *Precision* and *Recall*. *mAP@0.5* denotes mean average precision across all detection categories at an intersection-over-union threshold of 0.5 and serves as the primary indicator of overall detection and localization performance.

In line with common practice in real-time and underwater object detection, *mAP@0.5* is retained as the primary comparison metric, providing a clear and consistent decision rule for ranking the models. Because *mAP@0.5* alone is a relatively lenient localization criterion for multi-target, occluded, and small-object detection, we additionally introduce the stricter, COCO-style *mAP@0.5:0.95*, which averages AP over IoU thresholds from 0.50 to 0.95 in steps of 0.05, as a complementary localization metric. Model comparison is therefore based on a comprehensive assessment rather than on a single lenient criterion: *mAP@0.5* provides the primary ranking, *mAP@0.5:0.95* characterizes performance under a more demanding localization criterion, under which ULFD-YOLO does not lead (Section 3.1), and *Precision*, *Recall*, and *F1-score* describe the detection behavior.

Deployment efficiency was quantified using Parameters (M), FLOPs (G), and Model Size (MB). Model Size denotes the weights-only file size saved using the same serialization procedure for every compared detector. FLOPs in Table 2, Table 3, Table 4, Table 5 and Table 6 were calculated for a 640 × 640 model input. The Jetson inference latency reported in Section 3.4 was measured at 448 × 640 to preserve the deployment camera-stream aspect ratio; the FLOP and latency values should therefore not be interpreted as measurements at the same input dimensions.

Uncertainty estimation. Because a single fixed hold-out partition was used rather than k-fold cross-validation, the uncertainty attached to ULFD-YOLO’s absolute performance estimate was quantified by non-parametric image-level bootstrap resampling of the 681-image held-out subset. In each of B = 1000 replicates, 681 images were drawn with replacement, and *mAP@0.5* and *mAP@0.5:0.95* were recomputed from the stored ground-truth annotations and model predictions. The 95% confidence interval was defined by the 2.5th and 97.5th percentiles of the bootstrap distribution. Resampling was performed at the image level so that all detections belonging to the same image remained together.

For a direct comparison of the primary metric, a paired image-level bootstrap was performed for ULFD-YOLO and YOLOv11n using the same 1000 resampled image-index sets for both detectors. For each replicate, the difference Δ = *mAP@0.5*_ULFD-YOLO − *mAP@0.5*_YOLOv11n was calculated, and the 95% percentile bootstrap confidence interval of Δ was obtained. The number of bootstrap replicates yielding a non-negative difference was also recorded. This paired design preserves the dependence created by evaluating both detectors on the same images and distinguishes model-to-model differences from variation caused by the finite evaluation sample.

Strict-localization performance was additionally compared at the category level using a two-sided Wilcoxon signed-rank test on the paired *mAP@0.5:0.95* values of the 21 Fish-BJ detection categories. The test treats categories as the paired observational units and does not assume normally distributed differences. Training repeatability was summarized descriptively as the mean ± sample standard deviation across the primary run and the two additional training seeds. These analyses complement, but do not replace, validation on alternative dataset partitions.

## 3. Results

### 3.1. Comparison with Representative Detectors

The compared models were selected to provide a controlled and transparent benchmark for evaluating the accuracy–efficiency trade-off of ULFD-YOLO. In addition to high-complexity reference detectors, we included multiple nano-scale YOLO variants because they are widely used lightweight baselines with open implementations, stable training pipelines, and clear computational profiles. Although several recent underwater or fish-specific lightweight detectors have been proposed, direct numerical comparison is often complicated by differences in datasets, annotation protocols, input resolutions, hardware platforms, acceleration settings, and code availability. Therefore, this study focused on representative detectors evaluated under the controlled protocol described in Section 2.3. The reference implementations for Faster R-CNN, EfficientDet, DETR, YOLOv12, YOLOv13, and YOLO26 are described in [36,37,38,39,40,41]. Each model was trained and evaluated separately on Fish-BJ and on the selected WildFish subset using the corresponding fixed training/evaluation split. The quantitative results are summarized in Table 2.

To complement the mAP@0.5 comparison under a stricter localization criterion, Table 2 also reports mAP@0.5:0.95 for all detectors on both Fish-BJ and WildFish. Under this stricter criterion, ULFD-YOLO obtained 0.732 on Fish-BJ and 0.553 on WildFish. The ultra-lightweight model does not lead on mAP@0.5:0.95: YOLOv11n (0.848) and YOLOv8n (0.842) retain more precise box localization because the aggressive backbone compression that produces the 1.3 M-parameter footprint also sacrifices fine localization accuracy. ULFD-YOLO nevertheless retains a high mAP@0.5 of 0.960 while reducing the parameters and computation of YOLOv11n by 50.0% and 58.7%, respectively. Its mAP@0.5:0.95 is 0.031 lower than YOLOv3-tiny (0.763) and 0.043 lower than YOLOv13n (0.775), making the localization cost explicit rather than describing the scores as equivalent. The subsequent component-level ablation results show that adding the hypergraph neck to the compressed backbone raises mAP@0.5:0.95 from 0.723 to 0.775, recovering part of the strict-localization performance lost during backbone compression.

Image-level bootstrap resampling of the 681-image held-out Fish-BJ subset (B = 1000; Section 2.4) yielded mAP@0.5 = 0.960 (95% CI: 0.946–0.973) and mAP@0.5:0.95 = 0.732 (95% CI: 0.638–0.821) for ULFD-YOLO. The narrower interval for mAP@0.5 indicates that the primary metric is estimated more precisely than the strict-localization metric on this finite evaluation subset. These intervals describe evaluation-set sampling uncertainty and should not be interpreted as training-seed variability.

The paired image-level bootstrap supported the conclusion that ULFD-YOLO has a small but statistically detectable reduction in the primary metric relative to YOLOv11n: the recomputed bootstrap point estimates were 0.959 for ULFD-YOLO and 0.977 for YOLOv11n, giving ΔmAP@0.5 = −0.018 with a 95% percentile bootstrap confidence interval of −0.026 to −0.010. All 1000 paired bootstrap differences were negative. The paired bootstrap used the released prediction-level AP recomputation script; small differences from the framework-native point estimates in Table 2 may arise from differences in AP interpolation and aggregation as well as table rounding. Under the stricter localization criterion, the two-sided Wilcoxon signed-rank test across the 21 Fish-BJ categories gave mean category-wise mAP@0.5:0.95 values of 0.735 for ULFD-YOLO and 0.848 for YOLOv11n, with a median paired difference of −0.111 (W = 0, *p* < 0.001). The category-wise mean is an equal-weight average across categories and therefore need not exactly equal the dataset-level value in Table 2. Together, the analyses support the conclusion that the baseline retains higher localization accuracy, particularly at high IoU thresholds.

The statistical difference should be interpreted together with deployment cost. In the primary run, ULFD-YOLO reduced mAP@0.5 by 0.020 and mAP@0.5:0.95 by 0.116 relative to YOLOv11n, while reducing the parameter count from 2.6 M to 1.3 M and FLOPs from 6.3 G to 2.6 G. The contribution is therefore an accuracy–efficiency operating point rather than accuracy superiority: the proposed model accepts a measurable localization cost in exchange for 50.0% fewer parameters and 58.7% less computation.

The repeated-seed experiments supported the repeatability of this interpretation under the fixed split. Across model-training seeds 0, 1, and 42, ULFD-YOLO achieved mAP@0.5 = 0.969 ± 0.008 and mAP@0.5:0.95 = 0.754 ± 0.019, whereas YOLOv11n achieved 0.979 ± 0.001 and 0.847 ± 0.001, respectively. The mAP@0.5 gap ranged from 0.005 to 0.020, and the mAP@0.5:0.95 gap ranged from 0.080 to 0.116. The ranking and central accuracy–efficiency trade-off were therefore repeatable across the three training seeds under the unchanged data partition, although ULFD-YOLO showed greater seed sensitivity under the stricter localization metric.

Checkpoint sensitivity was limited for the main accuracy metrics. Evaluating the final-epoch ULFD-YOLO checkpoint (last.pt), rather than the framework-generated best.pt checkpoint, yielded Precision = 0.928, Recall = 0.884, mAP@0.5 = 0.958, mAP@0.5:0.95 = 0.728, and F1-score = 0.905. Relative to best.pt, the decreases were only 0.002 for mAP@0.5 and 0.004 for mAP@0.5:0.95. This sensitivity check suggests that the default validation-based checkpoint selection had only a minor effect on the reported mAP point estimates, although the held-out subset still cannot be regarded as a fully independent test set.

To characterize category-level behavior, Table 3 reports the Precision, Recall, mAP@0.5, and mAP@0.5:0.95 of ULFD-YOLO across the 21 Fish-BJ detection categories, together with the total number of annotated instances in the complete retained dataset. Per-category mAP@0.5 ranges from 0.913 for Spotted Scat to 0.995 for Speckled Pavon, whereas mAP@0.5:0.95 ranges from 0.654 for Tobies and Spotted Scat to 0.849 for Metynnis hypsauchen. Because the instance-count column aggregates the training and held-out subsets, it is provided as descriptive dataset context rather than as the effective sample size for each category-level evaluation metric. Detailed per-category and per-split image and bounding-box counts are provided in the public repository. The variation in strict-localization performance is consistent with the qualitative failure cases discussed in Section 3.3.1.

#### 3.1.1. Comparison with High-Complexity Reference Detectors

Faster R-CNN and DETR are included as representative high-complexity reference points, not as architecture-specifically optimized upper bounds. On Fish-BJ, Faster R-CNN achieved 0.958 mAP@0.5 with 28.4 M parameters, 37.6 GFLOPs, and a 108.4 MB weights-only model file, whereas DETR achieved 0.920 mAP@0.5 with 41.3 M parameters, 190.4 GFLOPs, and a 474.1 MB weights-only model file. By comparison, ULFD-YOLO achieved 0.960 mAP@0.5 with 1.3 M parameters, 2.6 GFLOPs, and a 3.0 MB weights-only model file. These results illustrate the substantially lower deployment cost of the proposed model under the reported equal-budget comparison, while no claim is made that it exceeds separately optimized versions of the high-complexity detectors.

From an aquaculture engineering perspective, the model size and computational cost of Faster R-CNN and DETR exceed the capacity of many embedded systems used in farms. Models requiring more than 100 MB of storage or tens to hundreds of GFLOPs per frame are difficult to accommodate within the memory, power, and thermal budgets of underwater camera nodes or edge modules installed in ponds, cages, or recirculating aquaculture systems (RAS). The 36-fold reduction in model size achieved by ULFD-YOLO relative to Faster R-CNN (3.0 MB vs. 108.4 MB) directly addresses this constraint.

#### 3.1.2. Comparison with Lightweight YOLO Variants

Among the nano-scale YOLO detectors, the YOLOv11n baseline achieved the highest Fish-BJ mAP@0.5 (0.980) with 2.6 M parameters and 6.3 GFLOPs. ULFD-YOLO reduced these values to 1.3 M parameters and 2.6 GFLOPs (−50.0% and −58.7%) while maintaining 0.960 mAP@0.5, a decrease of only 0.02 in absolute terms. Compared with YOLOv5n (0.977 mAP@0.5, 1.8 M parameters, and 4.2 GFLOPs), ULFD-YOLO uses 27.8% fewer parameters and 38.1% fewer FLOPs while maintaining competitive accuracy. Relative to YOLOv8n, ULFD-YOLO achieves higher mAP@0.5 (0.960 vs. 0.951) with 56.7% fewer parameters and 67.9% fewer FLOPs. Similar trends are observed for YOLOv10n, YOLOv12n, YOLOv13n, and YOLO26n.

These comparisons indicate that ULFD-YOLO occupies a favorable position on the accuracy–efficiency Pareto frontier. This is important for aquaculture monitoring scenarios in which multiple camera nodes must operate simultaneously on shared edge computing infrastructure with limited aggregate computational capacity.

#### 3.1.3. Performance on a Second Dataset After Dataset-Specific Training

ULFD-YOLO was trained and evaluated using the WildFish-specific split, a second dataset with a different category composition and more heterogeneous imaging conditions. As shown in Table 2, most detectors obtained lower values on the selected WildFish subset than on Fish-BJ, indicating that the second dataset is more challenging under the adopted protocol. ULFD-YOLO achieved 0.887 Precision, 0.698 Recall, 0.803 mAP@0.5, and 0.781 F1-score on WildFish. These results show that the architecture remains computationally efficient and retains useful detection capability after dataset-specific training under a different image distribution; they do not constitute a zero-shot transfer test from Fish-BJ.

These results should also be interpreted in relation to deployment cost. Although YOLOv5n and Faster R-CNN achieved higher WildFish mAP@0.5 values (0.947 and 0.922, respectively), they require 1.8 M and 28.4 M parameters and 4.2 and 37.6 GFLOPs, respectively. For strictly constrained embedded platforms, ULFD-YOLO provides a favorable efficiency–performance trade-off. Because every detector was trained on the WildFish training split before evaluation, this experiment should be interpreted as evaluation on a second dataset after dataset-specific training rather than evidence of zero-shot transfer. The result shows that the deployment-oriented accuracy–efficiency behavior can also be observed after training under the second dataset distribution.

### 3.2. Ablation Studies

#### 3.2.1. Backbone Comparison

To justify the selection of the custom MobileNetV4-tiny configuration, the original YOLOv11n backbone was replaced with several representative alternatives, including heavyweight (U-NetV2) [42], medium-scale (LSKNet, GhostNetV3, and UniRepLKNet) [43,44,45], and lightweight architectures (ShuffleNetV1, FasterNet, StarNet, MobileNetV4-Small, and the custom MobileNetV4-tiny configuration) [20,31]. All models were trained under identical conditions, and the results are summarized in Table 4.

The results reveal a clear trade-off between representational capacity and deployment efficiency. U-NetV2 matched the baseline accuracy (0.98 mAP@0.5) but required 15.4 M parameters and 37.2 GFLOPs, far exceeding the computational budget of embedded aquaculture devices. Among the lightweight candidates, MobileNetV4-tiny achieved 0.95 mAP@0.5 with only 1.8 M parameters, 4.5 GFLOPs, and a 3.9 MB model size, making it the most computationally efficient competitive backbone. Although StarNet and ShuffleNetV1 achieved slightly higher mAP@0.5 values (0.97 and 0.96), their efficiency advantages were weaker. Compared with ShuffleNetV1, MobileNetV4-tiny reduced parameters by 43.8%, FLOPs by 10.0%, and model size by 42.6%, with only a 0.01 decrease in mAP@0.5. These results support MobileNetV4-tiny as the backbone for the proposed deployment-oriented detector.

#### 3.2.2. Feature Fusion Network Analysis

To evaluate the effectiveness of the Hyper-YOLO neck, several alternative feature fusion strategies were tested with the MobileNetV4-tiny backbone, including BiFPN, DySample, EUCB, and FreqFusion. The results are shown in Table 5.

Among all candidates, Hyper-YOLO achieved the best overall balance between detection accuracy and model compactness, reaching 0.96 mAP@0.5, 0.93 Precision, 0.92 Recall, and 0.92 F1-score with only 1.6 M parameters, 3.7 GFLOPs, and a 3.5 MB model size. Compared with BiFPN, Hyper-YOLO improved mAP@0.5 by 0.01, Recall by 0.04, and F1-score by 0.03, with only 0.5 M additional parameters and 0.3 additional GFLOPs. The Recall improvement is particularly relevant to aquatic monitoring because it is consistent with better preservation of cues from small and partially occluded fish than conventional bidirectional pyramid fusion. In downstream stock-assessment applications, fewer missed detections may reduce the risk of undercounting, although counting accuracy was not evaluated directly in this study.

DySample showed severe performance degradation (0.69 mAP@0.5 at 37.5 GFLOPs), indicating that greater computation does not necessarily produce effective underwater feature fusion. EUCB was compact in parameter count (0.47 M) but required 15.8 GFLOPs and achieved only 0.800 mAP@0.5, suggesting that excessive simplification weakens semantic integration. FreqFusion achieved reasonable accuracy (0.94 mAP@0.5) but required 9.4 M parameters and 36.5 GFLOPs, which conflicts with the lightweight design objective. These results confirm that Hyper-YOLO is accurate and structurally well suited to the proposed ultra-lightweight detector.

#### 3.2.3. Component-Level Ablation

To quantify the individual and combined contributions of the proposed modules, a full factorial ablation study was performed. The MobileNetV4-tiny backbone (M), Hyper-YOLO neck (H), and Detect_MBConv head (D) were introduced individually and in combination. The results are reported in Table 6.

Individual contributions. Each component has a distinct functional role when applied alone. The M-only configuration achieved the strongest compression, reducing the model to 1.8 M parameters and 4.5 GFLOPs with 0.950 mAP@0.5, confirming its role as the efficiency-oriented foundation. The H-only configuration preserved near-baseline accuracy (0.980 mAP@0.5) but increased complexity to 3.2 M parameters and 8.1 GFLOPs, indicating its role as a representation enhancement module. The D-only configuration achieved 0.977 mAP@0.5 with 2.3 M parameters and 5.1 GFLOPs, demonstrating that it reduces prediction-stage cost with minimal accuracy loss.

Pairwise interactions. The combinations reveal complementary effects. M+H improved mAP@0.5 from 0.950 to 0.960 while further reducing parameters to 1.6 M and FLOPs to 3.7 G, confirming that Hyper-YOLO compensates for the representational limitations of the lightweight backbone. H+D achieved the highest mAP@0.5 among all configurations (0.982) with 2.9 M parameters and 6.5 GFLOPs, showing strong complementarity between enhanced feature fusion and efficient prediction. M+D yielded the strongest parameter compression (1.1 M parameters and a 2.6 MB model size) while maintaining 0.957 mAP@0.5, but at a higher computational cost than the complete model (3.0 versus 2.6 GFLOPs). This point is revisited below in relation to the Hyper-YOLO neck.

The observation that H+D attains a higher mAP@0.5 (0.982) than the complete M+H+D model (0.960) is consistent with the ablation and does not contradict the final design choice. H+D retains the original YOLOv11n backbone, preserving near-baseline accuracy but requiring 2.9 M parameters and 6.5 GFLOPs, more than twice the computation of the final model. The reduction from H+D to M+H+D is therefore attributable to MobileNetV4-tiny, which is the dominant source of both compression and the accuracy trade-off. M+D is smaller in parameters (1.1 M versus 1.3 M) but requires more computation (3.0 versus 2.6 GFLOPs) and attains a slightly lower mAP@0.5 (0.957 versus 0.960). The complete M+H+D configuration therefore represents a Pareto-efficient accuracy–computation trade-off and provides the lowest-FLOP operating point among the tested configurations while retaining 0.960 mAP@0.5. H+D remains a valid option when peak accuracy is prioritized, whereas M+D remains a valid option when parameter count or storage is the binding constraint.

M+D attains 1.1 M parameters and 0.957 mAP@0.5, whereas the complete M+H+D model attains 1.3 M parameters and 0.960 mAP@0.5. The corresponding differences are small in absolute terms—0.003 in mAP@0.5, 0.003 in mAP@0.5:0.95, and 0.001 in Recall—but no direct paired significance test was performed between these two ablation variants. They are therefore described as numerically comparable rather than statistically indistinguishable. Their main distinction is computational allocation: M+D is smaller in parameters and storage (1.1 M and 2.6 MB) but requires 3.0 GFLOPs, whereas M+H+D requires 2.6 GFLOPs at 1.3 M parameters and 3.0 MB. Because the objective of this study is sustained real-time inference under a constrained power budget, the lower-FLOP M+H+D configuration was selected. M+D remains a valid alternative when parameter count or storage, rather than computation, is the binding constraint.

Full integration. The complete ULFD-YOLO model (M+H+D) achieved 1.3 M parameters, 2.6 GFLOPs, and a 3.0 MB model size while maintaining 0.960 mAP@0.5, 0.920 Precision, 0.910 Recall, and 0.915 F1-score. Relative to YOLOv11n (2.6 M parameters, 6.3 GFLOPs, and 0.980 mAP@0.5), this represents a 50.0% reduction in parameters and a 58.7% reduction in FLOPs, with only a 0.02 absolute decrease in mAP@0.5. This result supports the central design premise that coordinated redesign of the backbone, neck, and detection head can substantially compress the model while largely preserving fish detection performance in underwater aquaculture environments.

### 3.3. Qualitative Analysis

#### 3.3.1. Visual Comparison of Detection Results

**Figure 5:**
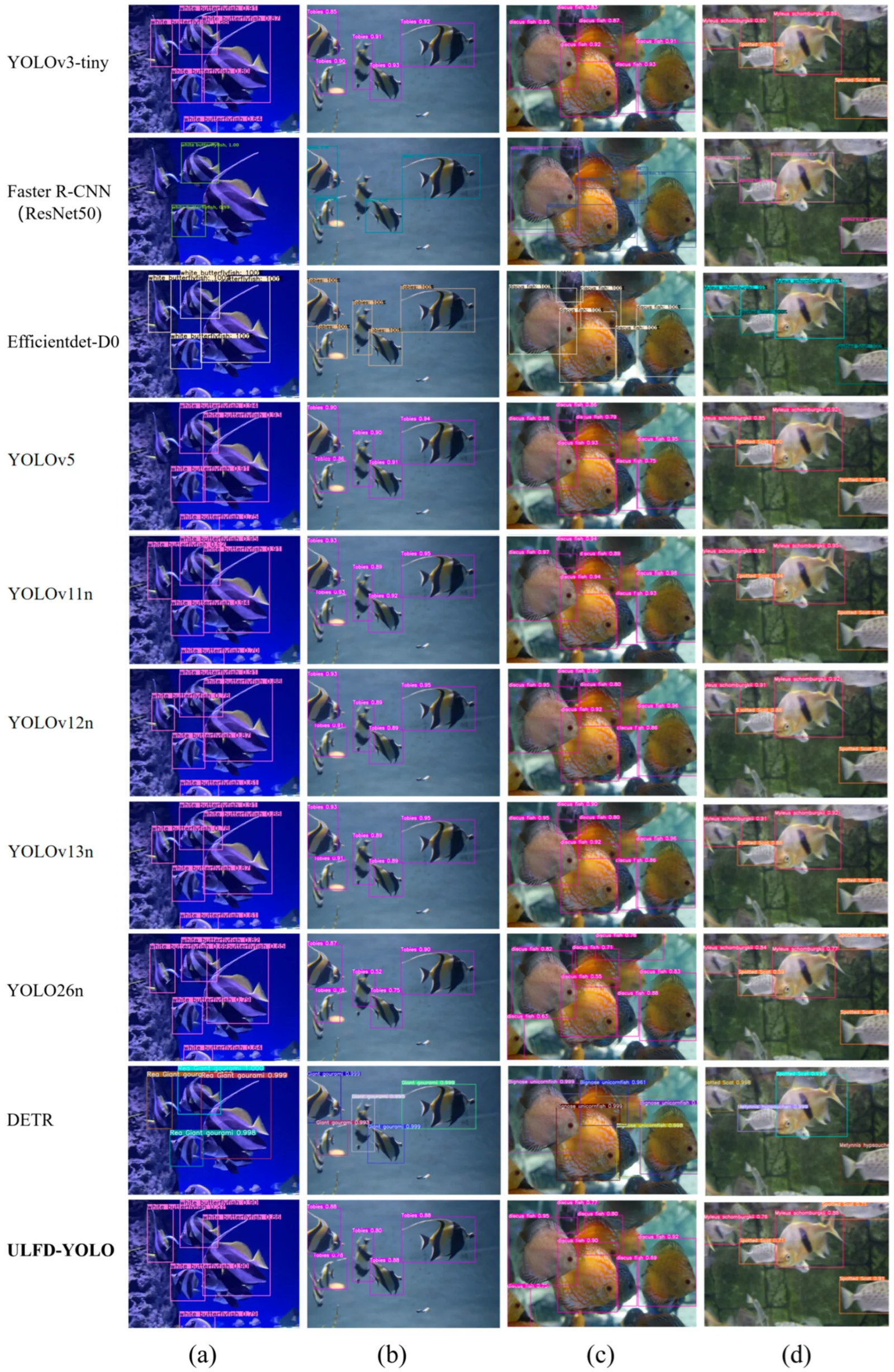
Qualitative comparison and failure-case analysis of detection results across representative underwater scenes. The upper rows show typical detection scenarios, including (**a**) multi-target scenes, (**b**) small and distant targets, (**c**) dense distributions with mutual occlusion, and (**d**) complex background interference. Green bounding boxes indicate correct detections. The bottom rows present representative failure cases, including missed detections of small low-contrast fish, false positives caused by background textures, localization errors or merged boxes under dense occlusion, and missed or duplicated detections under strong illumination gradients and color distortion. These examples illustrate both the practical detection capability and the remaining limitations of ULFD-YOLO under challenging underwater conditions. (Bounding-box and label colors are detector-generated visualization settings and do not represent additional experimental variables.).

To examine the practical detection behavior of ULFD-YOLO beyond quantitative metrics, qualitative comparisons were conducted against all compared detectors across representative underwater scenes. These scenes covered typical challenges in aquaculture monitoring, including multiple targets, small and distant fish, dense distributions with mutual occlusion, and cluttered backgrounds with aquatic vegetation and particulate matter. The visualization results are shown in Figure 5.

Traditional lightweight models, such as YOLOv3-tiny, showed weaker localization stability in dense scenes, with less compact predicted boxes when multiple fish were adjacent. Heavyweight models, including Faster R-CNN and DETR, produced stable detections but required substantially higher computational cost. Recent lightweight YOLO variants generally performed well, although their results varied in scenes with small or partially overlapping fish.

ULFD-YOLO maintained stable detection performance across the selected scenes. In the multi-target scene, it detected the major fish instances with clear localization boundaries, indicating that the lightweight redesign does not substantially weaken multi-target representation. In scenes containing small and spatially separated fish, the model captured the principal target instances, supporting the effectiveness of the backbone-neck co-design in preserving small-target information under compression. In the dense occlusion scenario, ULFD-YOLO produced detection boxes with relatively complete coverage, suggesting that the Hyper-YOLO neck helps separate semantically similar and spatially neighboring targets. This capability is directly relevant to monitoring densely stocked ponds and cage systems. Under complex background interference, the model continued to localize fish targets without obvious detection failure, indicating that the Detect_MBConv head retains sufficient discriminative capacity in cluttered underwater conditions.

To provide a balanced view, representative failure cases are also presented in Figure 5 (bottom rows). Four recurring error types were observed: (i) missed detections of small, distant fish with weak contrast against turbid water; (ii) false positives triggered by strongly textured backgrounds such as tank biofilm, plants, and suspended particles; (iii) localization errors and merged boxes under dense mutual occlusion, where neighboring individuals are grouped into a single detection; and (iv) missed or duplicated detections under strong illumination gradients and color distortion. These errors are consistent with the stricter mAP@0.5:0.95 result and with the reduced Recall observed on WildFish. They indicate that the residual limitations of the ultra-lightweight design are concentrated in the small-target and heavy-occlusion regimes rather than being uniformly distributed, which in turn identifies small-object feature preservation and occlusion-aware post-processing as the most promising directions for future improvement.

#### 3.3.2. Feature-Response Heatmap Analysis

**Figure 6:**
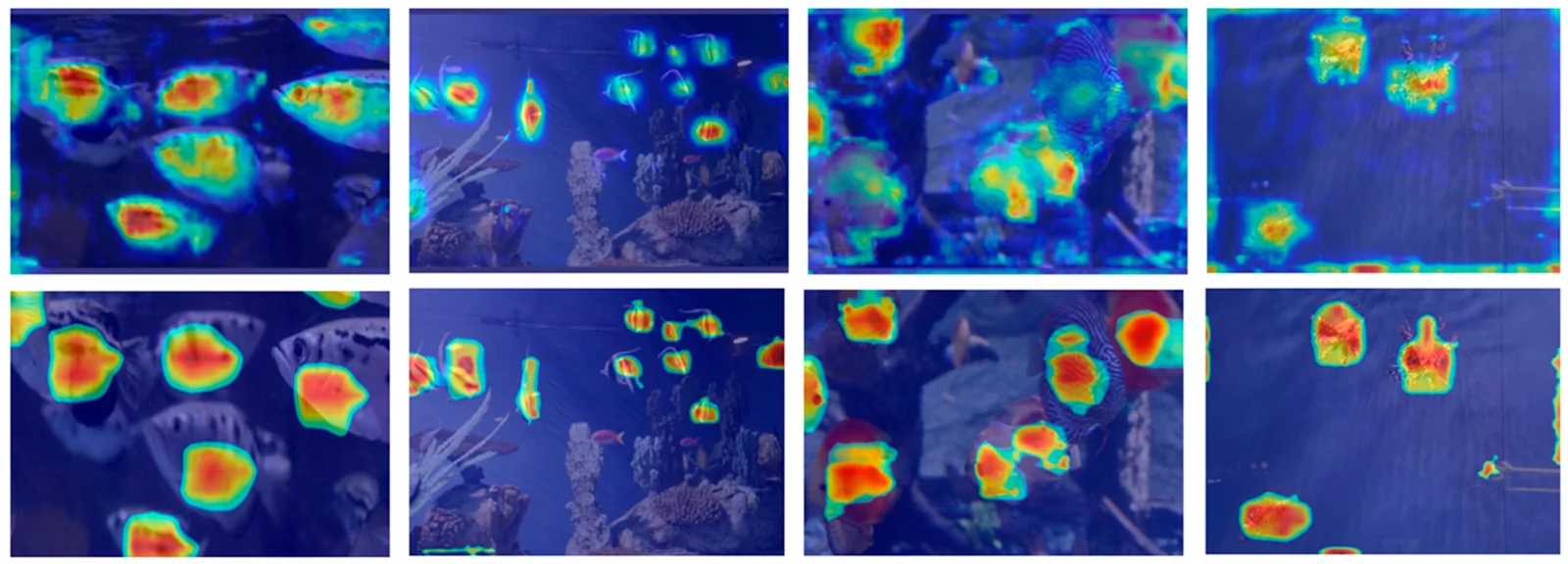
Grad-CAM heatmap comparison between the baseline detection head and the proposed Detect_MBConv head. Warmer colors indicate higher feature response intensity. Detect_MBConv produces more compact and target-centered activation regions, with improved suppression of irrelevant background areas, indicating enhanced spatial attention allocation for underwater fish detection.

To further analyze the effect of the modified detection head, Grad-CAM heatmap visualizations were generated for the baseline head and the proposed Detect_MBConv head across representative underwater scenes (Figure 6).

The modified head produced more compact and target-centered response regions than the baseline. In the baseline configuration, activated areas were more diffuse and sometimes extended into surrounding background regions. After replacing the original head with Detect_MBConv, high-response regions became more concentrated on fish body areas, indicating improved focus on task-relevant features during prediction. This pattern was particularly evident in scenes containing adjacent fish, where the modified head showed clearer separation of response regions among neighboring individuals. For small or distant targets, the modified head preserved recognizable activation hotspots, suggesting that the lightweight redesign did not severely weaken sensitivity to weak target cues. The baseline heatmaps also showed noticeable activation in non-target regions with strong texture or illumination variation, whereas the modified head showed lower response intensity in irrelevant areas. These qualitative results suggest that MBConv-based feature transformation and SE-based channel reweighting promote more effective spatial attention allocation under complex underwater conditions.

For reproducibility, the Grad-CAM maps in Figure 6 were computed at the final feature layer feeding the detection head (the last neck output stage before prediction), and the gradients were taken with respect to the fish class score of the highest-confidence detection in each image. The same layer and target were used for the baseline head and the Detect_MBConv head to ensure a fair visual comparison.

### 3.4. Edge Deployment Validation

To verify practical deployability on embedded aquaculture hardware, ULFD-YOLO was tested on an NVIDIA Jetson Orin Nano Developer Kit (NVIDIA Corporation, Santa Clara, CA, USA) with 8 GB RAM, a representative embedded artificial intelligence platform increasingly used in agricultural and aquacultural automation. The hardware and software configuration is summarized in Table 7.

The trained ULFD-YOLO model was deployed on the Jetson Orin Nano at FP32 precision with a batch size of 1. No TensorRT-based FP16 or INT8 quantization was applied in this experiment. The input resolution was 448 × 640 pixels, preserving the aspect ratio of the deployment camera stream. After an initial warm-up caused by CUDA kernel initialization and memory allocation, the model inference latency reported by the deployed pipeline stabilized at approximately 42–53 ms per frame, corresponding to about 19–24 FPS. These values represent model inference timing rather than full camera-acquisition-to-display latency. FLOPs in Table 2, Table 3, Table 4, Table 5 and Table 6 were calculated at 640 × 640, whereas the Jetson latency was measured at 448 × 640. The result therefore demonstrates real-time inference feasibility under the stated deployment configuration without additional quantization.

The reported “15 W” refers to the configured device power mode of the Jetson Orin Nano, namely the nvpmodel power budget, rather than an independently measured total system power consumption obtained using an external power meter. Therefore, the reported FPS should be interpreted as the real-time detection performance of the model under the 15 W device power mode. This clarification is important because the value does not represent the total electrical power drawn by the full camera–device system during inference. Nevertheless, the result demonstrates that ULFD-YOLO can operate under a low-power embedded configuration, which is relevant for camera-based fish monitoring systems where computational resources, memory, and power budgets are constrained.

**Figure 7:**
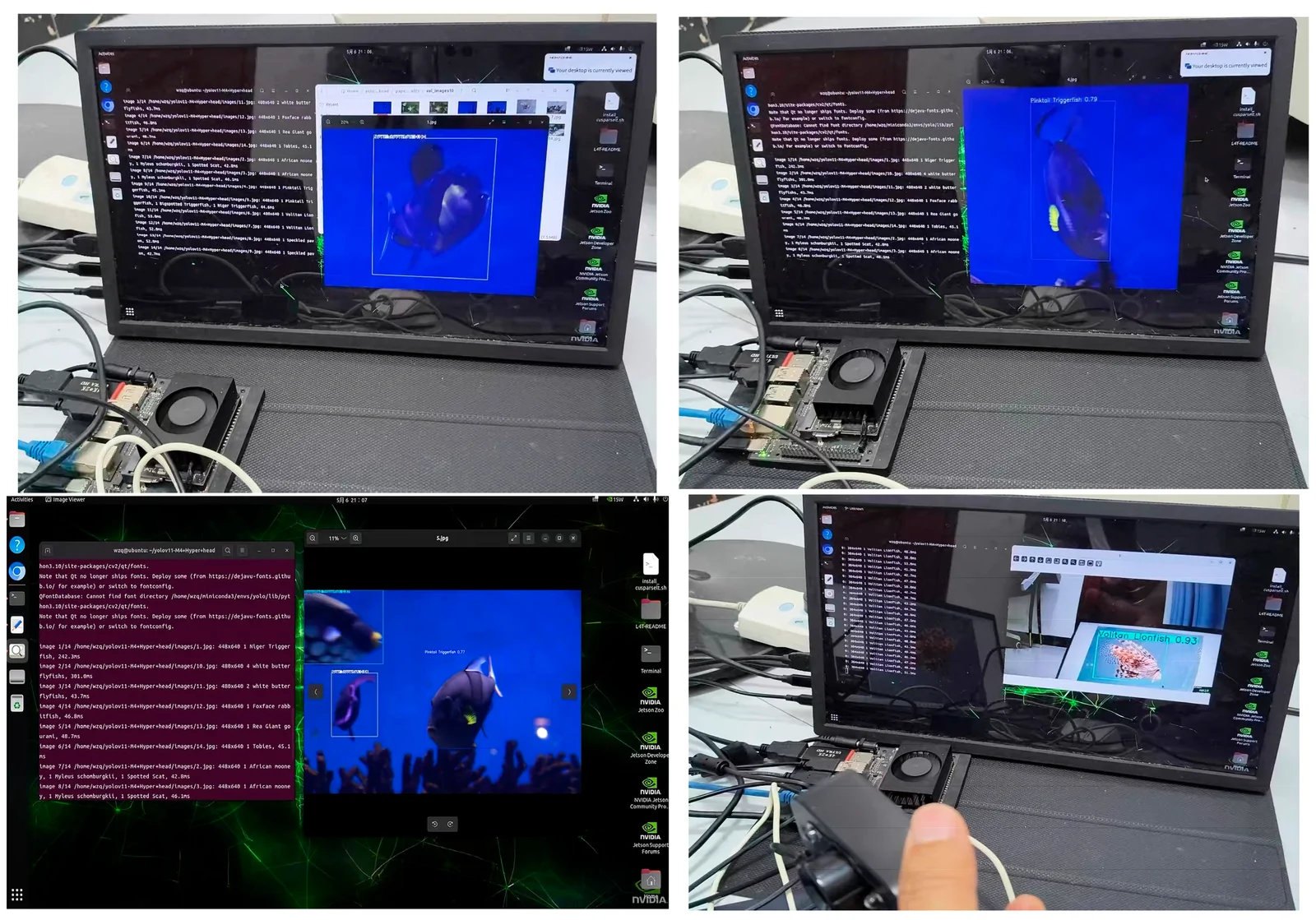
Edge deployment of ULFD-YOLO on the NVIDIA Jetson Orin Nano Developer Kit (NVIDIA Corporation, Santa Clara, CA, USA). The terminal output displays the model inference latency reported during camera-stream operation and the detected category, while the corresponding visualizations show bounding boxes with category labels and confidence scores. After warm-up, the model inference latency was approximately 42–53 ms per frame (19–24 FPS) at 448 × 640. This timing does not represent a separately measured full camera-acquisition-to-display pipeline.

Representative detection outputs are shown in Figure 7. These frames were selected from the camera-stream deployment to illustrate model behavior under edge-device operation. They are qualitative examples rather than the basis of the latency and FPS measurements. The compact weights-only model file and low computational cost allow ULFD-YOLO to run on the Jetson Orin Nano while retaining the ability to detect fish targets in underwater scenes.

This deployment experiment was conducted using the Jetson Orin Nano developer kit under controlled testing conditions. Although the external-camera test demonstrates real-time inference feasibility on embedded hardware, long-term field-level robustness still requires validation. The reported 42–53 ms value is the model inference latency shown by the deployed pipeline during sustained camera-stream operation; it does not include a separately measured camera-acquisition-to-display pipeline. We did not log the per-frame dispersion, decompose preprocessing, model inference, and post-processing times, benchmark the other lightweight detectors on the same device, or conduct multi-day field operation. A full embedded-systems characterization would additionally require thermal steady-state control, external power metering, and TensorRT-optimized variants. Accordingly, the deployment result is stated as evidence of real-time model inference under a specified configuration rather than as a complete system-throughput benchmark.

## 4. Discussion

### 4.1. Significance of the Proposed Approach for Aquaculture Monitoring

This study addresses a practical engineering challenge in automated aquatic animal monitoring. Reliable visual fish detection requires sufficient representational capacity to handle turbidity, low contrast, variable illumination, occlusion, and cluttered underwater backgrounds, whereas the embedded hardware available in aquaculture facilities imposes strict constraints on computation, memory, and power consumption. ULFD-YOLO is therefore best understood as a deployment-oriented redesign of YOLOv11n tailored to resource-constrained aquaculture monitoring platforms.

The experimental results support this design strategy. Compared with YOLOv11n, ULFD-YOLO reduces parameters from 2.6 M to 1.3 M and computational cost from 6.3 GFLOPs to 2.6 GFLOPs while maintaining 0.960 mAP@0.5 on Fish-BJ. This result shows that substantial compression can be achieved with a limited accuracy reduction when lightweight architectural design is combined with targeted feature compensation. The practical significance is clear for aquaculture facilities in which multiple camera nodes may share edge-computing infrastructure. The 2.6-GFLOP computational requirement and 3.0 MB storage footprint reduce the hardware barrier to future multi-camera and multi-stream deployment, although concurrent processing of several video feeds was not tested in this study.

### 4.2. Synergistic Co-Design of Backbone, Neck, and Detection Head

The ablation analysis shows that the three proposed modules are functionally complementary and that their coordinated integration is central to the final accuracy–efficiency balance. MobileNetV4-tiny provides the lightweight computational foundation by reducing backbone cost. Hyper-YOLO restores and strengthens multi-scale semantic representation through higher-order feature interaction. Detect_MBConv further reduces prediction-stage overhead while preserving discriminative capacity through channel-wise attention.

This finding provides a broader design insight for lightweight aquaculture detectors. Backbone compression alone can reduce computational cost, but it also weakens small-target representation. This weakness is critical in intensive aquaculture environments, where fish often appear at different distances from the camera, are partially obscured by neighboring individuals or farm structures, and are embedded in cluttered backgrounds. The Hyper-YOLO neck consistently improved Recall and F1-score under the lightweight setting, indicating that higher-order feature fusion helps recover difficult targets in crowded underwater scenes. Lightweight aquaculture detectors should therefore combine efficient backbone architectures with task-specific feature enhancement mechanisms that compensate for representational losses introduced by compression.

The Detect_MBConv head further demonstrates that the prediction stage offers an additional opportunity for computational savings when discriminative capacity is maintained through channel attention. Overall, the results support a co-design paradigm in which the backbone is optimized for efficiency, the neck for representational recovery, and the head for lightweight yet discriminative prediction.

The hypergraph neck models higher-order, multi-to-multi correlations: unlike an ordinary graph edge that connects two vertices, a hyperedge connects several vertices simultaneously, allowing semantically related fish cues distributed across spatial positions and semantic scales to be aggregated together. The isolated M-only versus M+H comparison supports this interpretation: Recall increases from 0.900 to 0.920 and mAP@0.5:0.95 from 0.723 to 0.775 when the hypergraph neck is added to the compressed backbone. The Grad-CAM analysis in Section 3.3.2 compares detection heads and is therefore interpreted only as qualitative evidence for Detect_MBConv, not as neck-specific evidence.

This comparison provides a plausible explanation for why the hypergraph neck performs better than the conventional necks under the tested configuration. PANet and BiFPN rely mainly on adjacent-layer pairwise feature propagation and have limited ability to model higher-order cross-scale and cross-position dependencies. The hypergraph neck instead constructs hyperedges that connect multiple semantically related vertices simultaneously, enabling global cross-location and cross-scale aggregation. This mechanism is consistent with the improved accuracy–compactness balance observed in Table 5, but the ablation does not establish it as the sole causal explanation.

### 4.3. Performance on a Second Dataset After Dataset-Specific Training and Implications for Multi-Site Deployment

The WildFish experiment provides additional evidence that the compact architecture can be trained and evaluated under a second dataset distribution. After dataset-specific training on the WildFish training split, ULFD-YOLO retained useful detection capability relative to models with larger computational budgets. This result is relevant to aquatic monitoring because deployments differ in category composition, water quality, illumination, and background structure across facilities, production cycles, and seasons.

The second-dataset result should not be interpreted as performance without retraining. Rather, it shows that the same compact architecture can be trained under a different dataset distribution while preserving a favorable accuracy–efficiency trade-off. In operational applications, some degree of site-specific calibration or fine-tuning may still be required, particularly when category composition and imaging conditions differ substantially from the development data.

The lower WildFish values (mAP@0.5: 0.803; Recall: 0.698) compared with the Fish-BJ results (0.960 and 0.910) indicate that the selected WildFish subset is substantially more difficult under the corresponding training and evaluation protocol. The Recall deficit is relevant to downstream stock-assessment applications because missed detections may contribute to undercounting, although counting accuracy was not evaluated directly in this study. Future work should therefore incorporate more heterogeneous multi-site training data, underwater-specific augmentation, and lightweight adaptation strategies to improve robustness across facilities and imaging regimes.

A qualitative inspection of the WildFish errors showed that missed detections were concentrated in images with high turbidity, strong blue-green color cast, cluttered natural backgrounds, small apparent target size, and partial occlusion. Large, well-illuminated, centrally placed fish were detected more reliably. Because these scene attributes were not annotated as formal strata, this analysis is descriptive rather than a quantitative subgroup test. Nevertheless, it identifies practical failure modes and motivates targeted remedies, including color and illumination augmentation, heterogeneous multi-site training data, and lightweight domain adaptation.

### 4.4. Limitations and Future Work

Several limitations should be acknowledged. First, although the Jetson Orin Nano experiment demonstrates real-time model-inference feasibility, validation was conducted under laboratory conditions rather than during long-term operation in a production facility. Field-level issues, including thermal management in waterproof enclosures, sensor–processor synchronization, long-duration stability, and integration with farm management software, require further verification. Second, this study focuses on still-image detection; temporal information and multi-object tracking were not explicitly modeled. Third, the on-device evaluation used a single camera-stream configuration and did not include complete pipeline timing, per-frame latency distributions, same-device baseline benchmarking, external power metering, or multi-day field testing. Fourth, the evaluation did not consider additional degradation caused by data transmission, such as compression artifacts and bandwidth limitations. Fifth, repeated training was conducted only for ULFD-YOLO and YOLOv11n and comprised three runs per model; this strengthens the principal comparison but does not characterize seed variability for every baseline and ablation variant. The bootstrap intervals quantify finite-evaluation-set uncertainty, whereas the Wilcoxon test uses only 21 detection categories as paired units; neither analysis substitutes for validation across multiple independent dataset partitions. Object-size-stratified AP was not reported because COCO area thresholds were not pre-specified for Fish-BJ; applying them retrospectively would require additional validation. Sixth, Fish-BJ was acquired with a camera operated in air outside the exhibit tanks and does not reproduce submerged-camera effects such as port refraction, lens biofouling, on-board-light backscatter, or low-light conditions at depth. Seventh, the held-out Fish-BJ subset served both for the Ultralytics default best-checkpoint selection and performance reporting, so it is not a fully independent test set. Eighth, visually difficult real-world scenes were intentionally retained; despite second-annotator review, a small degree of residual category or boundary uncertainty may remain for severely blurred, heavily occluded, or incompletely visible instances. Finally, the near-duplicate-aware split reduces the most direct leakage risk, but session, site, and individual-fish identifiers were not recorded; a fully leakage-controlled session- or individual-level evaluation was therefore not possible.

Future work will focus on six directions: (1) TensorRT-based FP16/INT8 quantization; (2) video-based temporal detection and multi-object tracking; (3) larger heterogeneous multi-site datasets and lightweight domain adaptation; (4) integration with automated stock counting, growth estimation, feeding optimization, and welfare assessment; (5) long-term field validation in operational aquaculture facilities; and (6) extension of multi-seed and paired statistical analysis to all baseline detectors and ablation variants, together with repeated evaluation under alternative train/evaluation partitions.

## 5. Conclusions

This paper presents ULFD-YOLO, an ultra-lightweight fish detection model redesigned from YOLOv11n for resource-constrained embedded aquaculture platforms. The architecture coordinates a custom convolutional MobileNetV4-tiny backbone, a hypergraph-based multi-scale fusion neck, and a lightweight Detect_MBConv prediction head. On Fish-BJ, the primary run achieved 0.960 mAP@0.5 (95% bootstrap CI: 0.946–0.973) with 1.3 M parameters, 2.6 GFLOPs, and a 3.0 MB weights-only model file, reducing parameters and computational cost by 50.0% and 58.7% relative to YOLOv11n. Across model-training seeds 0, 1, and 42, ULFD-YOLO achieved 0.969 ± 0.008 mAP@0.5 and 0.754 ± 0.019 mAP@0.5:0.95. After dataset-specific training on the difficult WildFish subset, the model achieved 0.803 mAP@0.5. Deployment on an NVIDIA Jetson Orin Nano sustained 19–24 FPS model inference at 448 × 640 under the platform’s 15 W nvpmodel power mode, demonstrating real-time inference feasibility under the tested configuration.

Overall, ULFD-YOLO provides a compact deployment-oriented solution for automated visual fish monitoring. Its principal contribution is not higher peak accuracy than YOLOv11n, but a detection–efficiency trade-off that was repeatable across three model-training seeds under the fixed data split. The paired bootstrap and category-level Wilcoxon analyses support the conclusion that YOLOv11n retains higher localization accuracy, while the repeated-seed experiments show that the relative ranking and efficiency advantage of ULFD-YOLO remain stable under the tested seeds. Future work should prioritize TensorRT optimization, video-based tracking, multi-site robustness, integration with downstream management tasks, repeated-partition validation, and long-term field testing.

The key quantitative findings are as follows: (1) coordinated backbone–neck–head redesign reduced parameters and computation by 50.0% and 58.7% relative to YOLOv11n; (2) the paired bootstrap estimated a primary-metric difference of −0.018, with a 95% percentile bootstrap confidence interval of −0.026 to −0.010 and all 1000 paired differences negative, while the category-level Wilcoxon test supported lower strict-localization performance for ULFD-YOLO (W = 0, *p* < 0.001); (3) across model-training seeds 0, 1, and 42, ULFD-YOLO achieved 0.969 ± 0.008 mAP@0.5, compared with 0.979 ± 0.001 for YOLOv11n, showing that the accuracy–efficiency trade-off was repeatable under the fixed split; (4) the final-epoch last.pt checkpoint differed from the framework-generated best.pt checkpoint by only 0.002 in mAP@0.5 and 0.004 in mAP@0.5:0.95; and (5) the model sustained 19–24 FPS model inference at 448 × 640 under the Jetson Orin Nano’s 15 W nvpmodel power mode. The compact footprint may facilitate future multi-camera monitoring networks, although concurrent multi-stream operation was not tested.

## Figures and Tables

**Table 1:** Summary of the Fish-BJ and WildFish datasets used in this study.

Datasets	Fish-BJ	WildFish
Model input size	640 × 640 pixels	640 × 640 pixels
Training images	2721	944
Held-out evaluation images	681	236
Detection categories	21	10
Annotated boxes	6050	1269
Split type	Category-stratified, near-duplicate-aware 8:2 hold-out	Fixed 8:2 hold-out with manually added detection boxes

**Table 2:** Quantitative comparison of ULFD-YOLO with representative object detectors on the Fish-BJ and WildFish datasets. Faster R-CNN, DETR, and EfficientDet retained the architecture-specific default optimization configuration of their reference implementations, except that batch size was adjusted to available memory and the epoch budget was standardized to 300. They are included as equal-budget reference points rather than architecture-specifically optimized upper bounds; the primary controlled comparison is among nano-scale YOLO-family models trained within the common framework. (Boldface indicates the proposed model.).

Detector	Precision		Recall		mAP@0.5		mAP@0.5:0.95		F1-Score		Parameters (M)	FLOPs (G)	Model Size (MB)
	Fish-BJ	WildFish	Fish-BJ	WildFish	Fish-BJ	WildFish	Fish-BJ	WildFish	Fish-BJ	WildFish			
YOLOv3-tiny	0.954	0.861	0.931	0.845	0.972	0.901	0.763	0.551	0.942	0.853	12.1	18.9	23.2
Faster R-CNN (ResNet50)	0.949	0.926	0.922	0.861	0.958	0.922	0.760	0.599	0.935	0.892	28.4	37.6	108.4
EfficientDet-D0	0.913	0.797	0.788	0.768	0.913	0.797	0.749	0.603	0.846	0.782	3.9	2.4	15.1
YOLOv5n	0.960	0.936	0.940	0.893	0.977	0.947	0.804	0.690	0.950	0.914	1.8	4.2	3.9
YOLOv8n	0.961	0.903	0.958	0.837	0.951	0.905	0.842	0.623	0.959	0.869	3.0	8.1	6.3
YOLOv10n	0.918	0.751	0.916	0.650	0.966	0.759	0.770	0.482	0.917	0.697	2.7	8.3	5.8
YOLOv11n	0.972	0.921	0.940	0.765	0.980	0.879	0.848	0.614	0.956	0.836	2.6	6.3	5.5
YOLOv12n	0.949	0.860	0.942	0.687	0.979	0.827	0.790	0.535	0.945	0.764	2.5	5.8	5.2
YOLOv13n	0.947	0.872	0.909	0.811	0.969	0.875	0.775	0.598	0.928	0.840	2.5	6.2	5.2
YOLO26n	0.851	0.751	0.860	0.728	0.921	0.786	0.672	0.483	0.855	0.739	2.5	5.8	5.2
DETR	0.920	0.910	0.860	0.840	0.920	0.910	0.798	0.721	0.889	0.874	41.3	190.4	474.1
**ULFD-YOLO**	**0.920**	**0.887**	**0.910**	**0.698**	**0.960**	**0.803**	**0.732**	**0.553**	**0.915**	**0.781**	**1.3**	**2.6**	**3.0**

Note: Table 2 reports the primary-run results obtained under the protocol described in Section 2.3. Model Size is the weights-only file size obtained using a consistent serialization procedure. Parameters and FLOPs were evaluated at 640 × 640 input. Absolute bootstrap intervals, paired comparison with YOLOv11n, final-epoch checkpoint sensitivity, and three-run repeatability results are reported below.

**Table 3:** Per-category detection performance of ULFD-YOLO on the 21 Fish-BJ detection categories and total annotated instances in the complete retained dataset.

Category	Precision	Recall	mAP@0.5	mAP@0.5:0.95	Instances (All Splits)
Pinktail Triggerfish	0.976	0.961	0.989	0.815	154
Bigspotted Triggerfish	0.866	0.946	0.982	0.770	186
Niger Triggerfish	0.930	0.811	0.935	0.713	249
Speckled Pavon	0.984	1.000	0.995	0.780	226
Tilapia buttikoferi	0.980	0.901	0.958	0.688	307
White Butterflyfish	0.944	0.929	0.975	0.694	393
Red Giant Gourami	0.919	0.948	0.977	0.811	265
Foxface rabbitfish	0.980	0.934	0.983	0.704	285
Orange-shoulder Tang	0.913	0.857	0.923	0.665	241
Tobies	0.791	0.939	0.930	0.654	403
Giant Gourami	0.917	1.000	0.990	0.809	222
Discus Fish	0.826	0.960	0.945	0.732	473
Bignose Unicornfish	0.912	0.865	0.957	0.727	206
Blood Parrot	0.931	0.917	0.959	0.810	212
Volitan Lionfish	0.972	0.957	0.989	0.801	238
Striped Surgeonfish	0.940	0.813	0.915	0.681	208
African Mooney	0.888	0.883	0.924	0.696	342
Myleus schomburgkii	0.923	0.884	0.954	0.672	423
Spotted Scat	0.861	0.900	0.913	0.654	358
Metynnis hypsauchen	0.934	0.833	0.972	0.849	242
Archerfish	0.809	0.877	0.952	0.719	417
Overall	0.920	0.910	0.960	0.732	6050

**Table 4:** Backbone comparison results of YOLOv11n-based models on the Fish-BJ dataset. (Boldface indicates the introduced backbone configuration.)

Backbone	Precision	Recall	mAP@0.5	mAP@0.5:0.95	F1-Score	Parameters (M)	FLOPs (G)	Model Size (MB)
Baseline	0.972	0.940	0.980	0.848	0.956	2.6	6.3	5.5
U-NetV2	0.960	0.950	0.980	0.802	0.955	15.4	37.2	31.1
ShuffleNetv1	0.940	0.920	0.960	0.739	0.930	3.2	5.0	6.8
LSKNet	0.950	0.930	0.970	0.740	0.940	12.1	35.1	24.5
GhostNetV3	0.950	0.940	0.980	0.824	0.945	13.4	15.0	28.0
UniRepLKNet	0.940	0.940	0.970	0.775	0.940	12.5	31.2	25.5
FasterNet	0.940	0.930	0.970	0.759	0.935	4.6	10.5	9.6
StarNet	0.940	0.910	0.970	0.734	0.925	1.9	5.0	4.2
MobileNetV4-Small	0.890	0.910	0.950	0.733	0.900	4.03	6.5	8.5
**MobileNetV4-tiny**	**0.920**	**0.900**	**0.950**	**0.723**	**0.910**	**1.8**	**4.5**	**3.9**

**Table 5:** Comparison of different neck architectures for feature fusion on the Fish-BJ dataset. (Boldface indicates the introduced neck configuration.)

Model	Precision	Recall	mAP@0.5	mAP@0.5:0.95	F1-score	Parameters (M)	FLOPs (G)	Model Size (MB)
BiFPN	0.910	0.880	0.950	0.698	0.895	1.1	3.4	2.6
DySample	0.680	0.650	0.690	0.335	0.665	1.7	37.5	3.9
EUCB	0.780	0.770	0.800	0.471	0.775	0.470	15.8	2.0
FreqFusion	0.910	0.890	0.940	0.749	0.900	9.4	36.5	18.3
**Hyper-YOLO**	**0.930**	**0.920**	**0.960**	**0.775**	**0.925**	**1.6**	**3.7**	**3.5**

**Table 6:** Ablation study of the proposed components in ULFD-YOLO on the Fish-BJ dataset. (“√” indicates that the component is included, whereas “×” indicates that the component is not included.)

Model	MobileNetV4	Hyper-YOLO	Detect_MBConv	Precision	Recall	mAP@0.5	mAP@0.5:0.95	F1-score	Parameters (M)	FLOPs (G)	Model Size (MB)
Baseline	×	×	×	0.972	0.940	0.980	0.848	0.956	2.6	6.3	5.5
M	√	×	×	0.920	0.900	0.950	0.723	0.910	1.8	4.5	3.9
H	×	√	×	0.970	0.950	0.980	0.816	0.960	3.2	8.1	6.8
D	×	×	√	0.976	0.946	0.977	0.843	0.961	2.3	5.1	4.9
M+H	√	√	×	0.930	0.920	0.960	0.775	0.925	1.6	3.7	3.5
H+D	×	√	√	0.956	0.957	0.982	0.840	0.956	2.9	6.5	6.1
M+D	√	×	√	0.910	0.909	0.957	0.735	0.909	1.1	3.0	2.6
M+H+D	√	√	√	0.920	0.910	0.960	0.732	0.915	1.3	2.6	3.0

**Table 7:** Hardware and software configuration of the edge deployment platform.

Item	Specification
Hardware platform	NVIDIA Jetson Orin Nano Developer Kit
SoC	Tegra234 (CUDA Arch 8.7)
Memory	8 GB LPDDR5
Operating system	Ubuntu 22.04 (L4T 36.5.0)
TensorRT	10.3.0.30 (installed; not used for the reported FP32 inference test)
Python	3.10.12

## Data Availability

A public repository associated with this study is available at https://github.com/999er/ULFD-YOLO (accessed on 22 June 2026). At the time of submission, the repository provides the exact Fish-BJ training/evaluation split list, per-category and per-split annotation statistics, the ULFD-YOLO model configuration and custom module implementation, training and validation scripts, prediction-export utilities, the stored held-out predictions, the outputs from the three training seeds, the statistical-analysis scripts, and the detection annotations created for the selected WildFish subset. The repository will be maintained and supplemented with additional implementation documentation as the release evolves. The Fish-BJ source images and trained model weights are available from the corresponding author upon reasonable request, subject to applicable institutional data-sharing conditions. The original WildFish source images remain publicly available from the dataset provider.
